# Supplementary material for: Mapping and characterising areas with high levels of HIV transmission in sub-Saharan Africa: A geospatial analysis of national survey data
Source: PLoS Med. 2020 Mar 6;17(3):e1003042. doi: 10.1371/journal.pmed.1003042 (PMC7059914; doi:10.1371/journal.pmed.1003042)
Supplement: S1 Equations — (DOCX) [file pmed.1003042.s002.docx]

**S1 Equations. Overview of equations utilised in the study.**

1. **Semivariogram model and ordinary kriging equations**

Exponential semivariogram model:

$$\gamma\left( h \right)=c_{0}+c\left( 1-\exp\left( \frac{-h}{a} \right) \right)$$

Where $h$ is the distance between any two locations $x_{1}$ and $x_{2}$, *c_0_* is the nugget, *c* is the partial sill and *a* is the range (in decimal degrees).

Ordinary kriging was performed to predict the logit-transformed outcome $Y$at unsampled locations with a spatial resolution of 5 km^2^:

$$Y\left( x_{i} \right)=logit\left( p\left( x_{i} \right) \right)$$

where $p\left( x_{i} \right)$ is the prevalence of HIV infection at location $x_{i}$

1. **Median odds ratio (MOR) equations**

Median odds ratio (MOR):

$\mathrm{MOR}=\exp[\surd((2*V\_a ) )*0.6745]\approx exp(0.95*\sqrt{\left( V_{a} \right)})$)

where ﻿*V_a_* is the area level variance, and 0.6745 is the 75th centile of the cumulative distribution function of the normal distribution with mean 0 and variance 1
