# Supplementary figures and images for: Mapping and characterising areas with high levels of HIV transmission in sub-Saharan Africa: A geospatial analysis of national survey data
Source: PLoS Med. 2020 Mar 6;17(3):e1003042. doi: 10.1371/journal.pmed.1003042 (PMC7059914; doi:10.1371/journal.pmed.1003042)

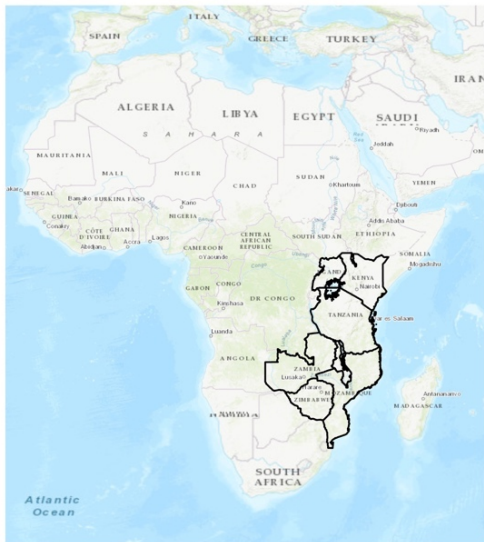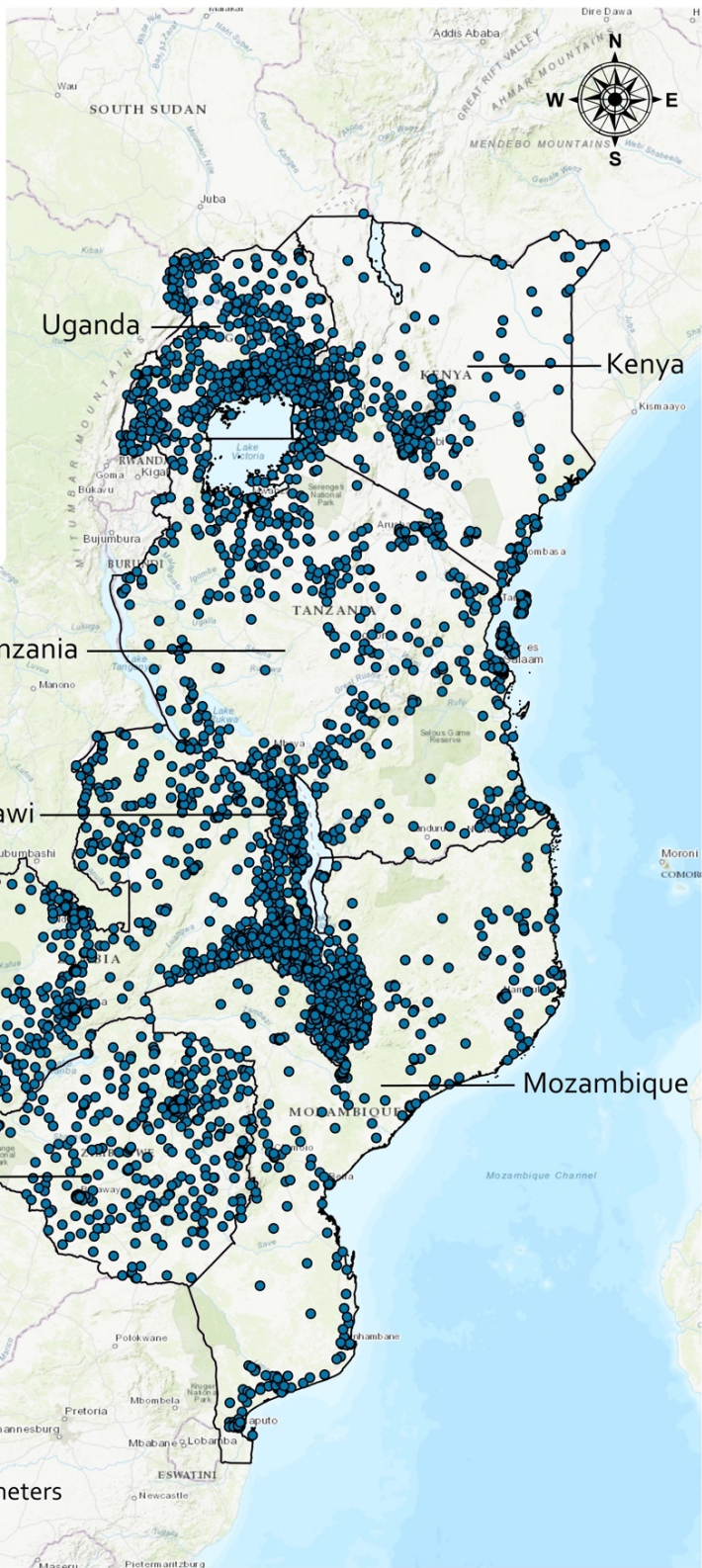

Supplement: S1 Fig — 1AIS. (PDF) [file pmed.1003042.s003.pdf]

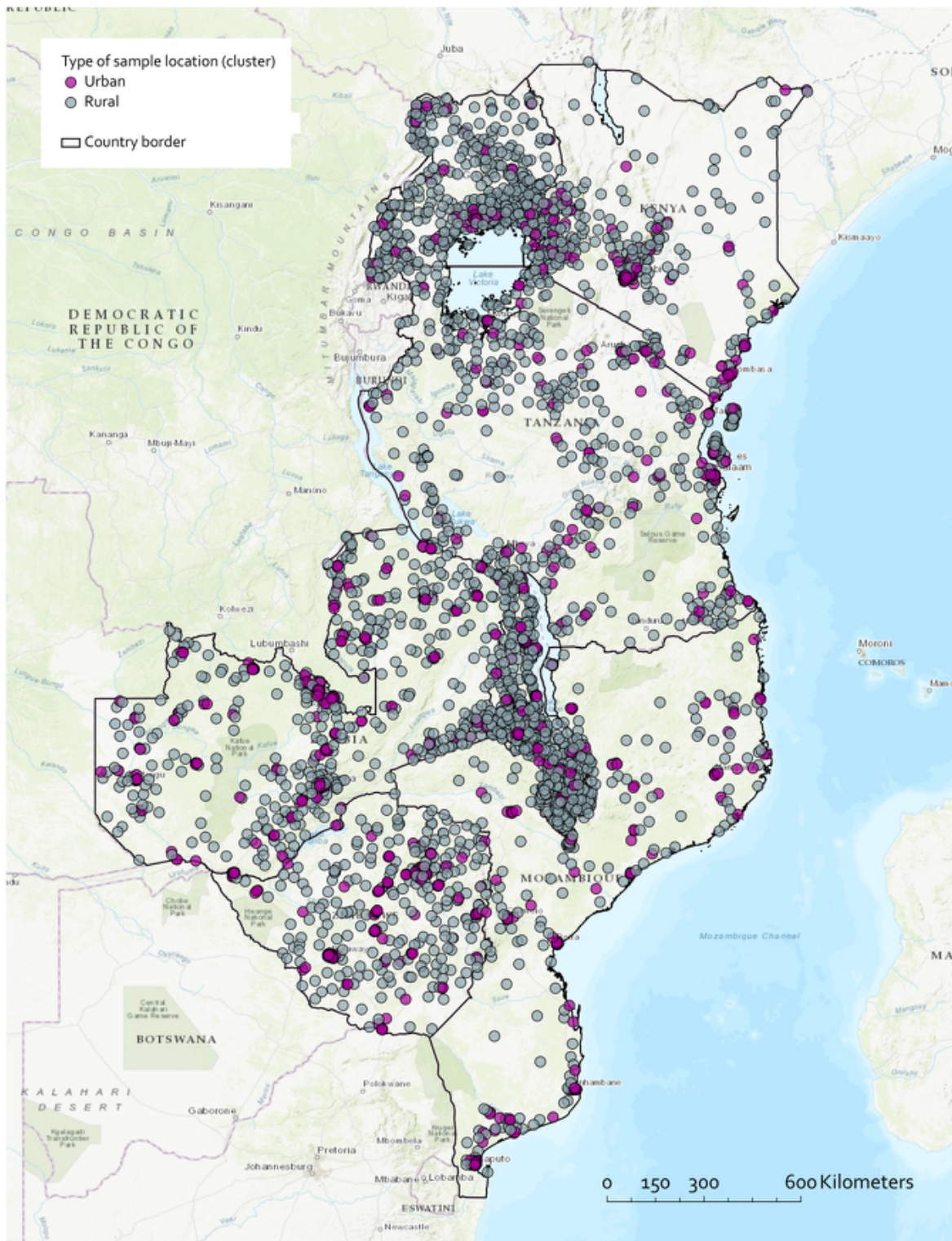

Supplement: S2 Fig — (PDF) [file pmed.1003042.s004.pdf]

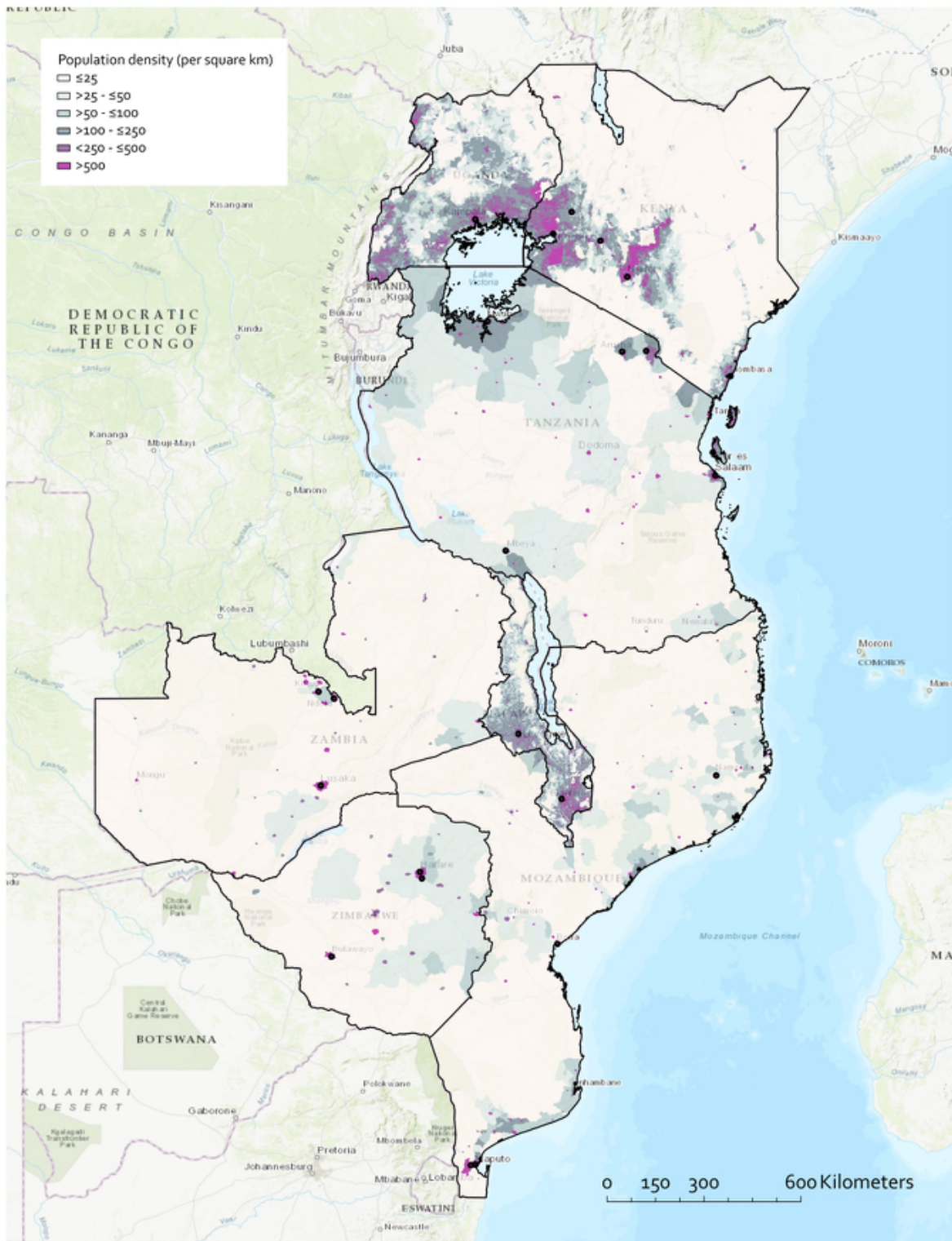

Supplement: S3 Fig — (PDF) [file pmed.1003042.s005.pdf]

Proximity to nearest highway (km)

- ≤5
- >5 - ≤10
- >10 - ≤50
- >50

□ Country border

— Highway

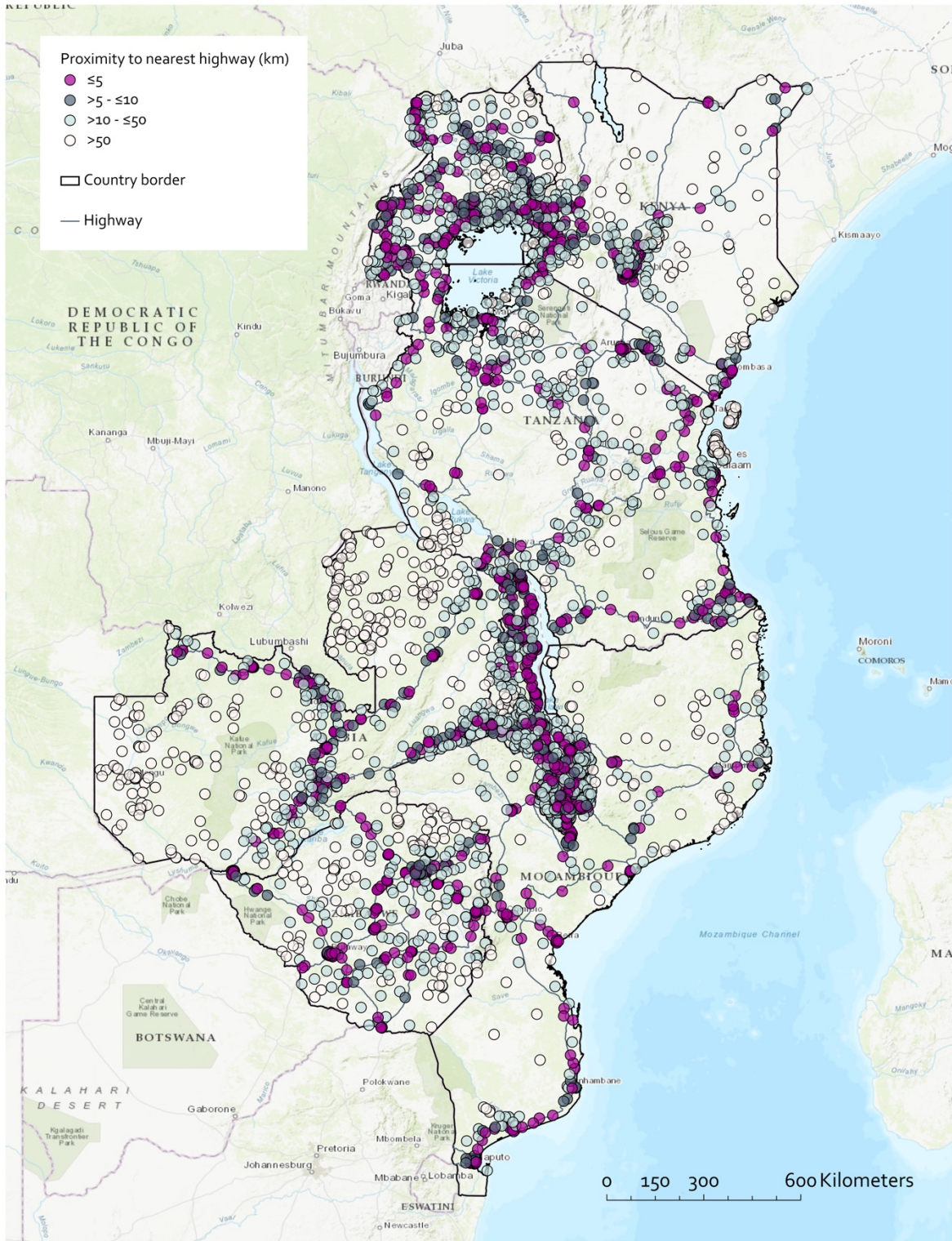

Supplement: S4 Fig — (PDF) [file pmed.1003042.s006.pdf]

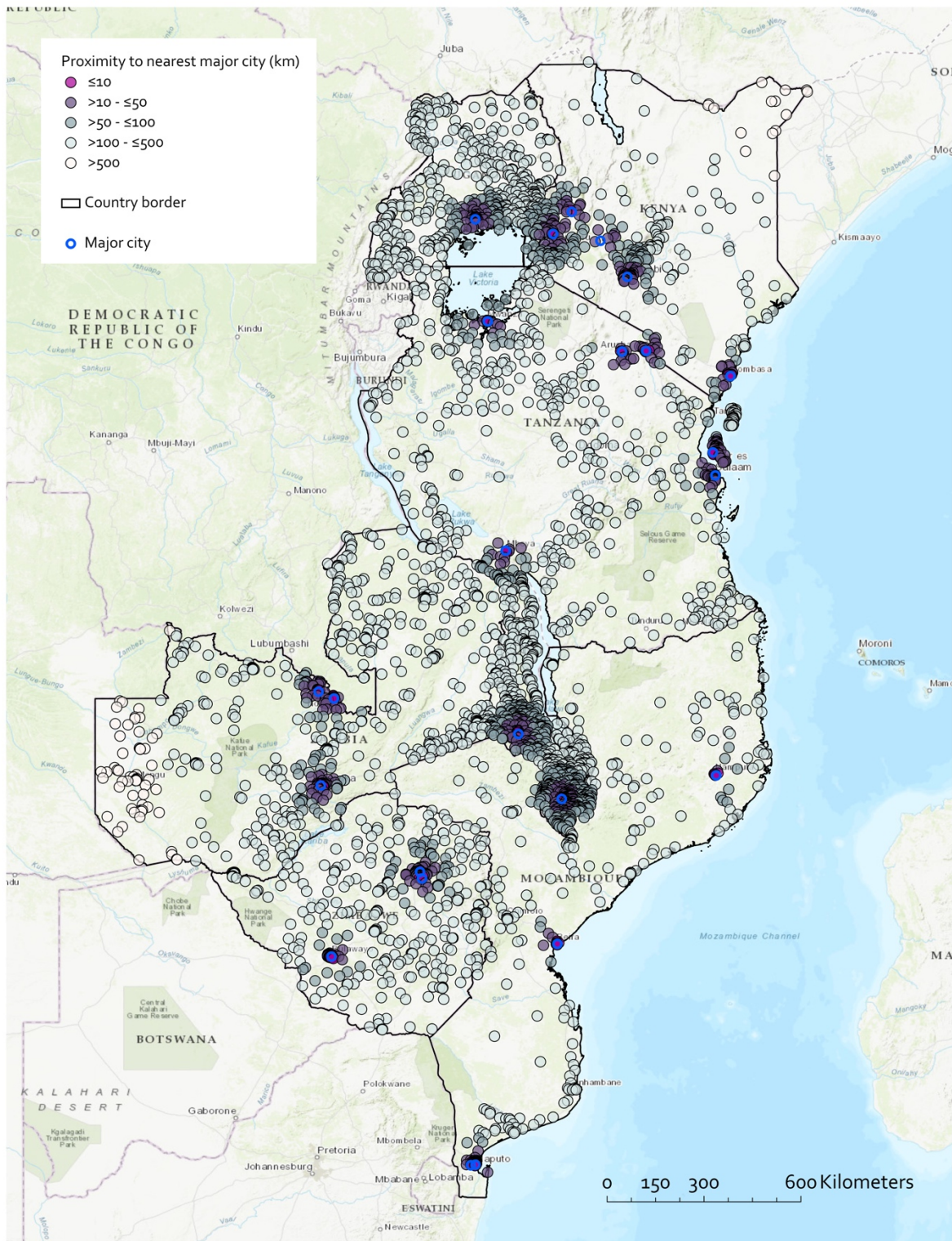

Supplement: S5 Fig — (PDF) [file pmed.1003042.s007.pdf]

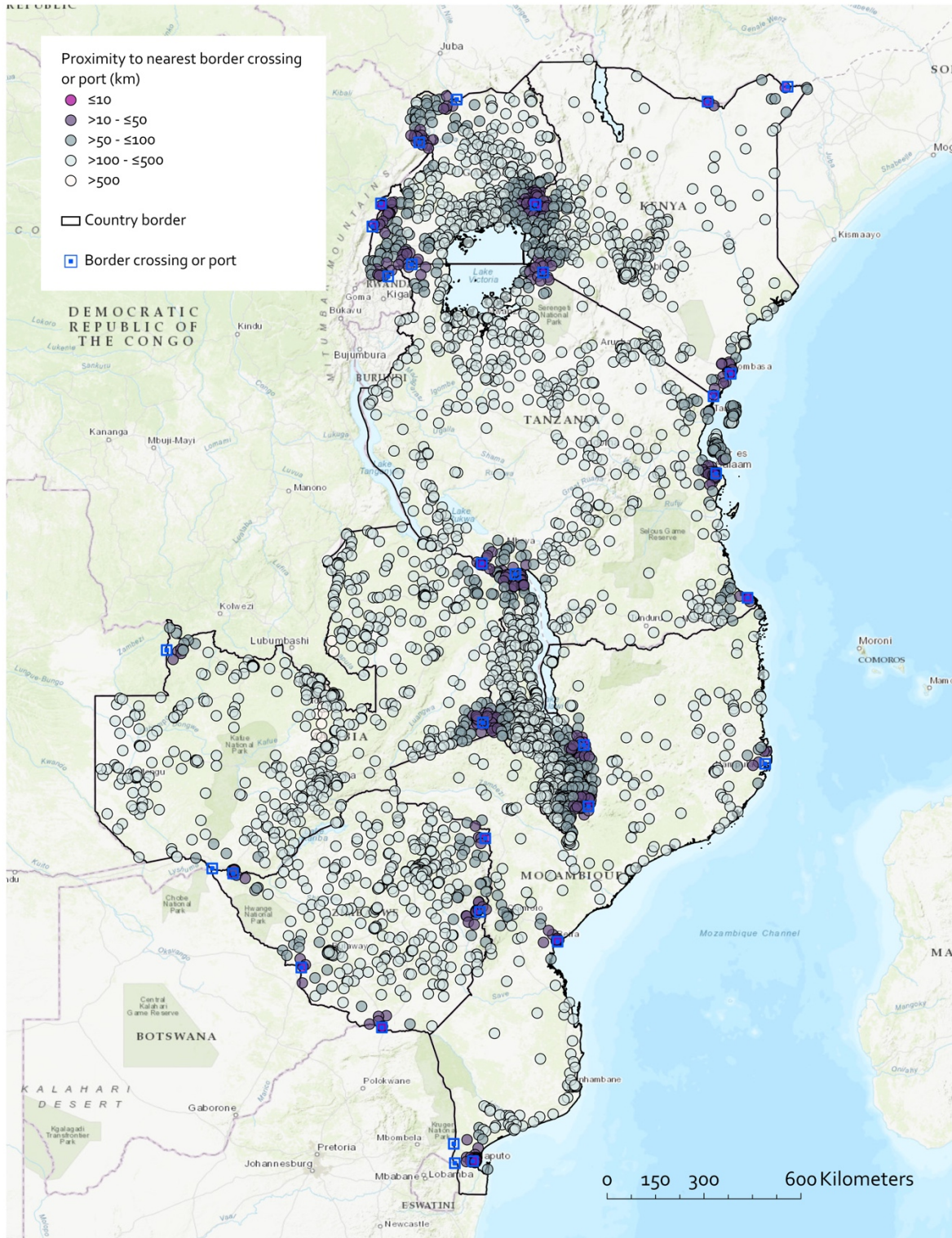

Supplement: S6 Fig — (PDF) [file pmed.1003042.s008.pdf]

### Enhanced Vegetation Index (EVI)

- ≤51
- >51 - ≤76
- >76 - ≤102
- >102 - ≤137
- >137 - ≤181
- >181 - ≤250

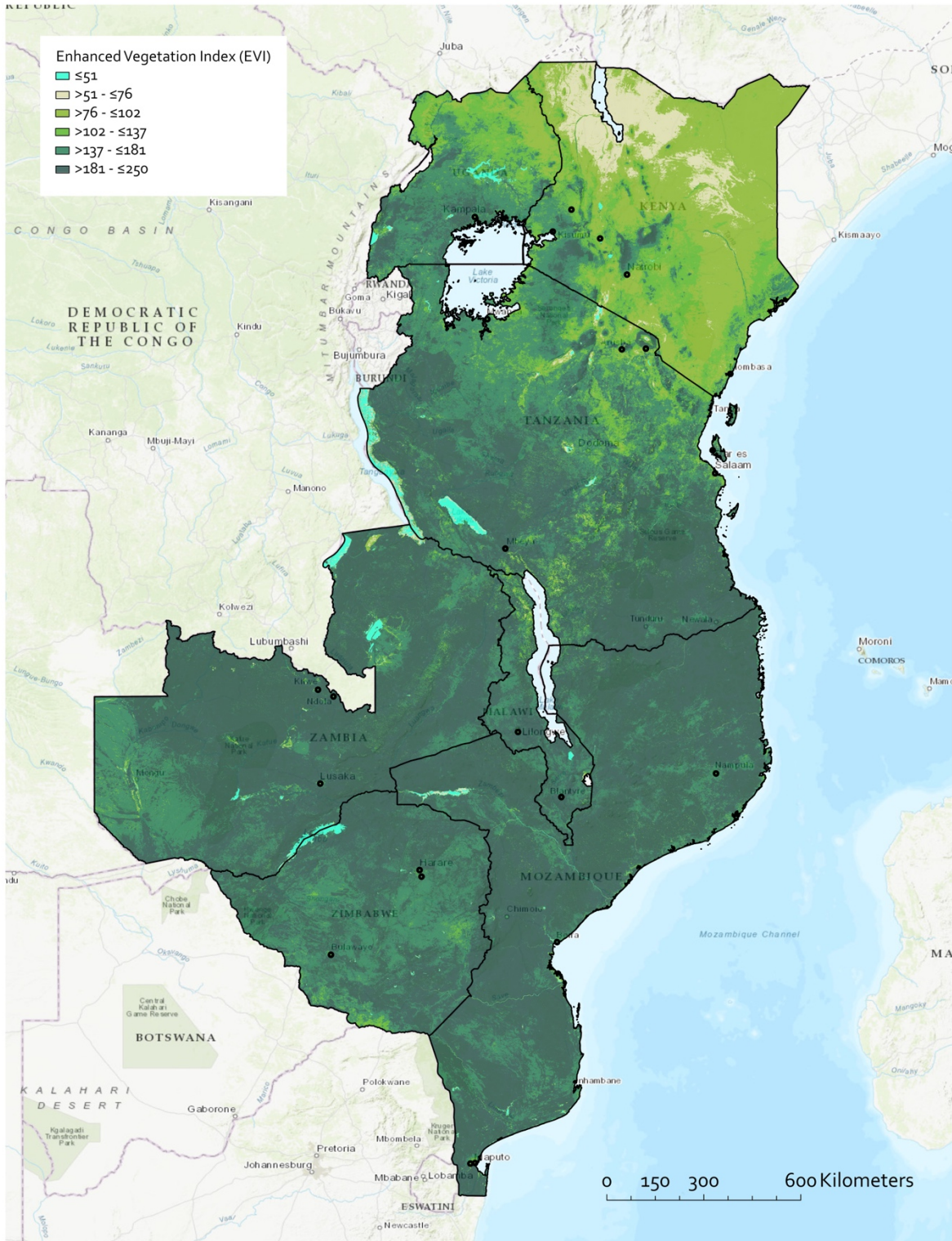

Supplement: S7 Fig — (PDF) [file pmed.1003042.s009.pdf]

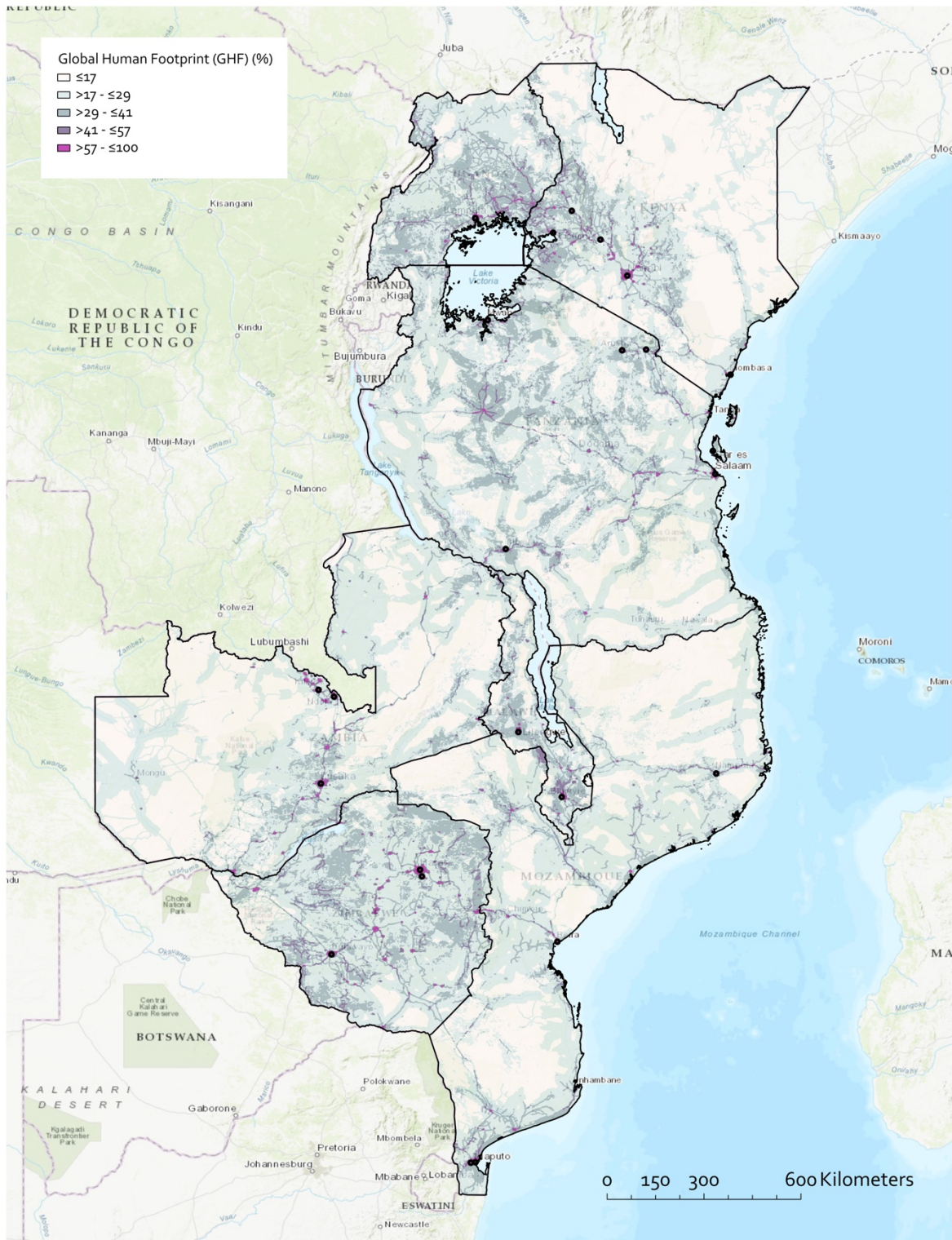

Supplement: S8 Fig — (PDF) [file pmed.1003042.s010.pdf]

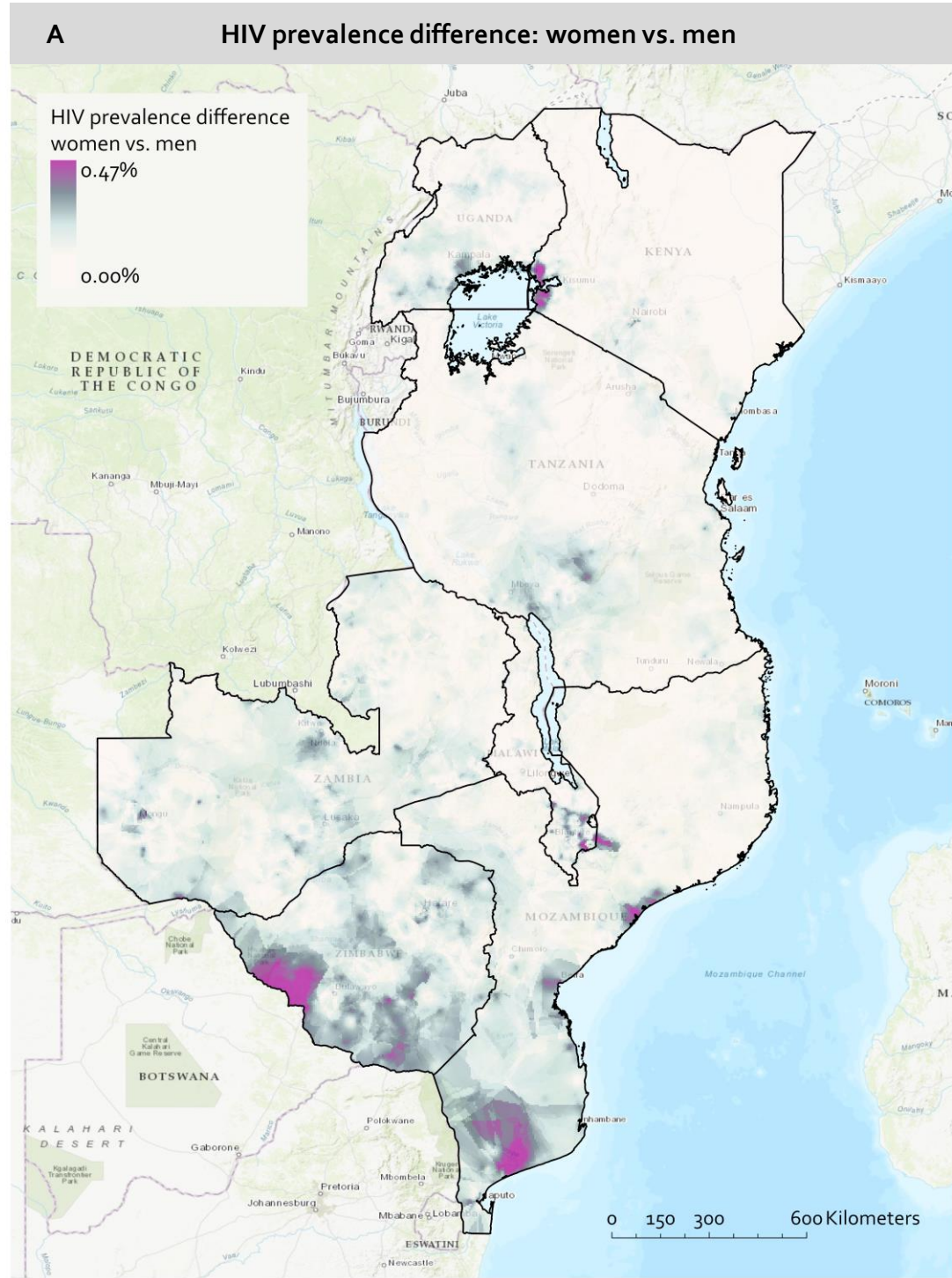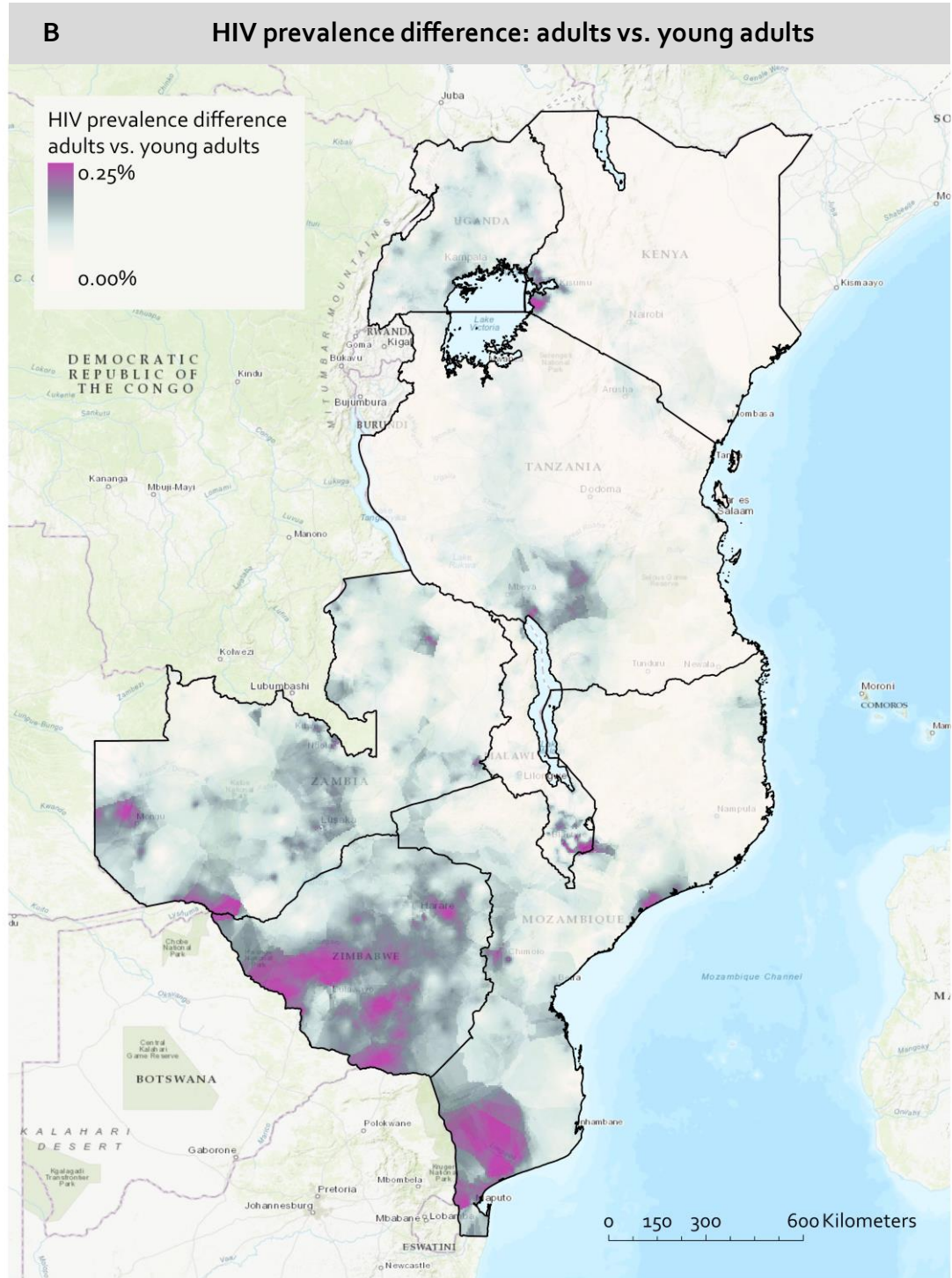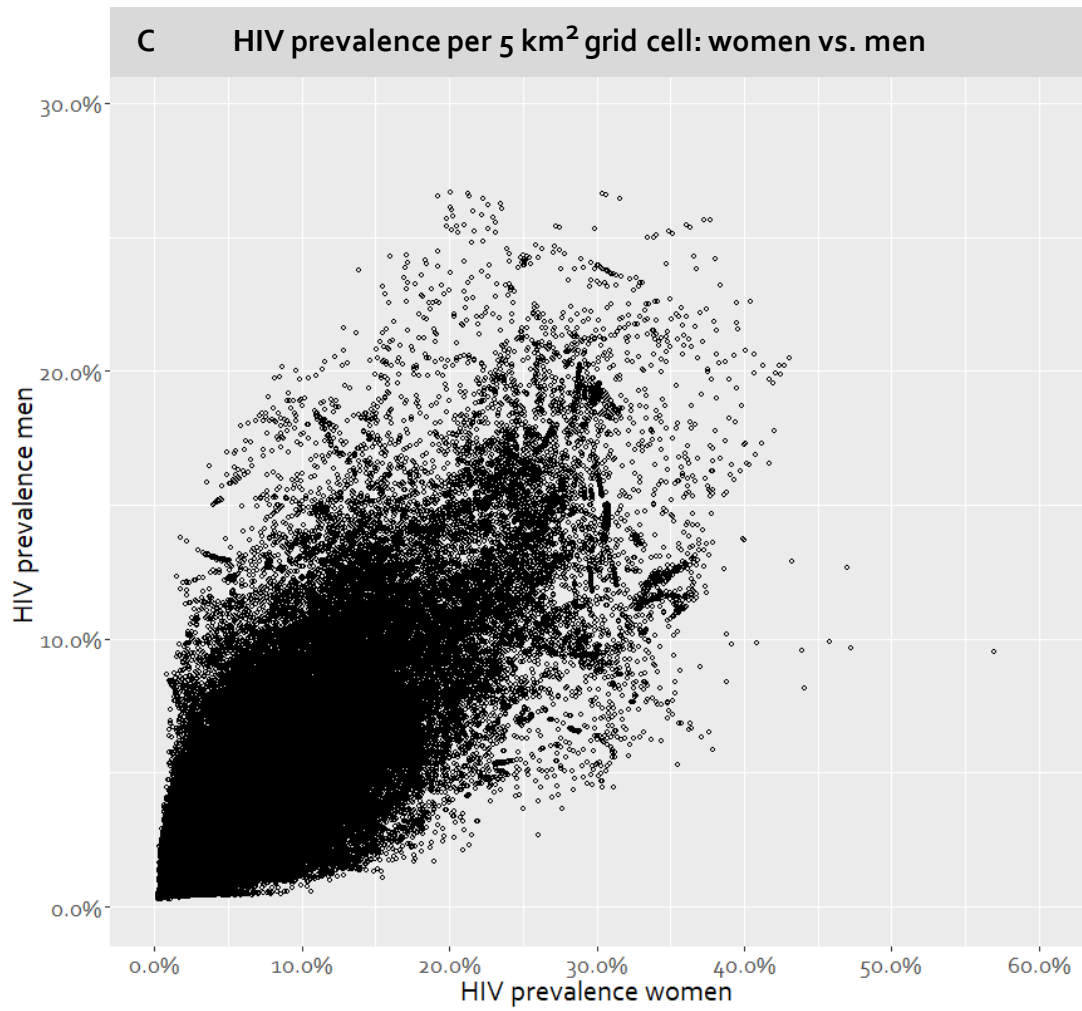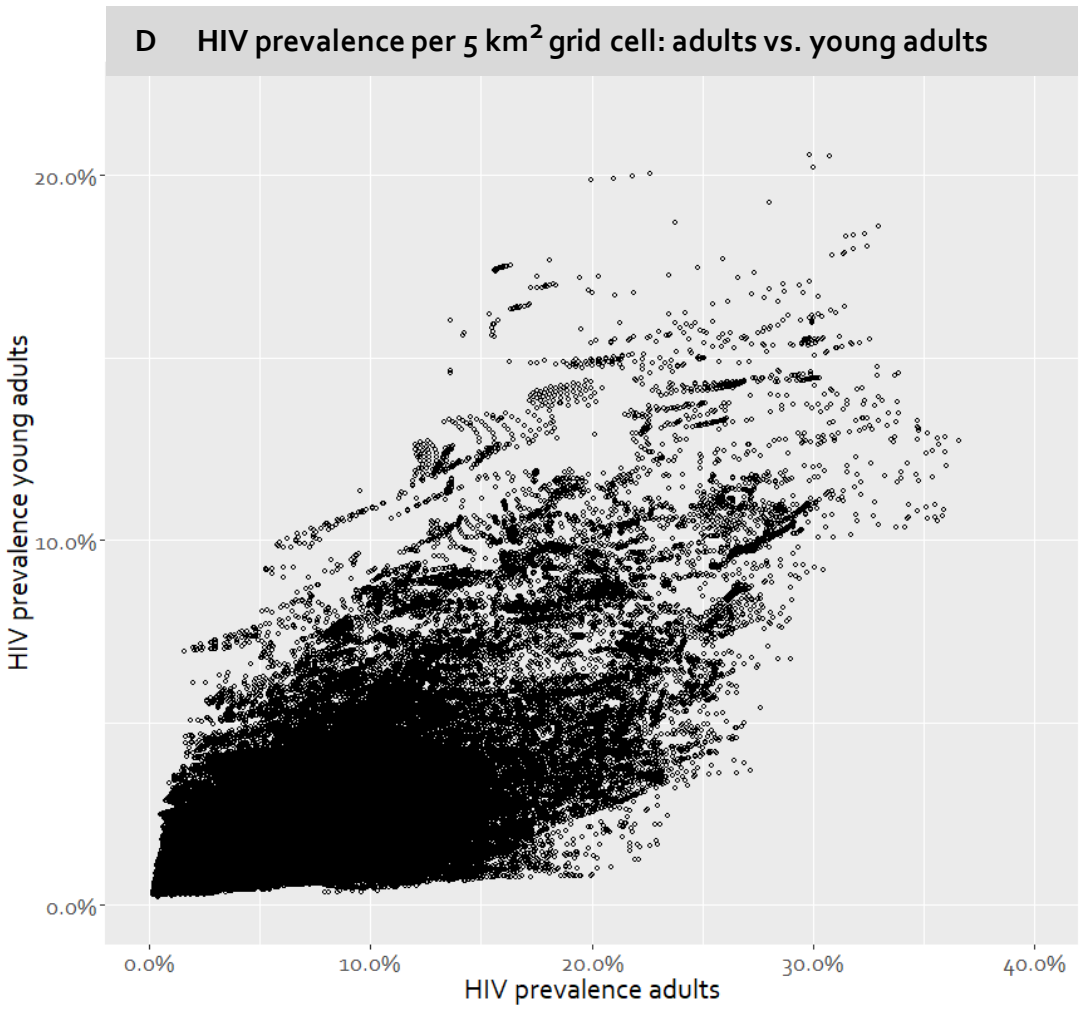

Supplement: S9 Fig — Maps present the predicted HIV prevalence in women (15–49 years) (A) and men (15–54 years) (B) for 7 countries in Eastern and Southern Africa. The maps of HIV prevalence among adults and young adults are shown in Fig 2. Continuous surface maps were created by kriging HIV prevalence data obtained from (https://dhsprogram.com/). (PDF) [file pmed.1003042.s011.pdf]

A

Women

HIV prevalence

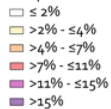

• Major city

— Highway

□ Country border

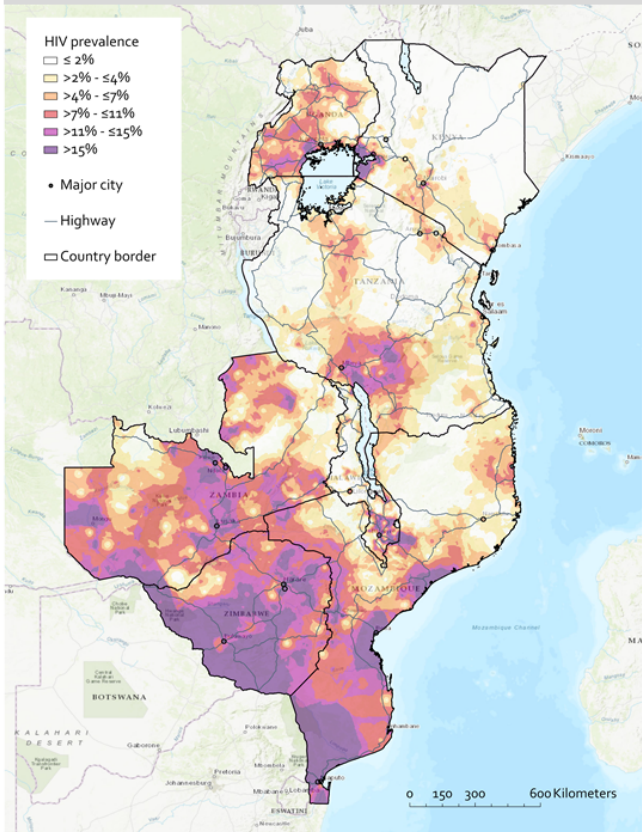

B

Men

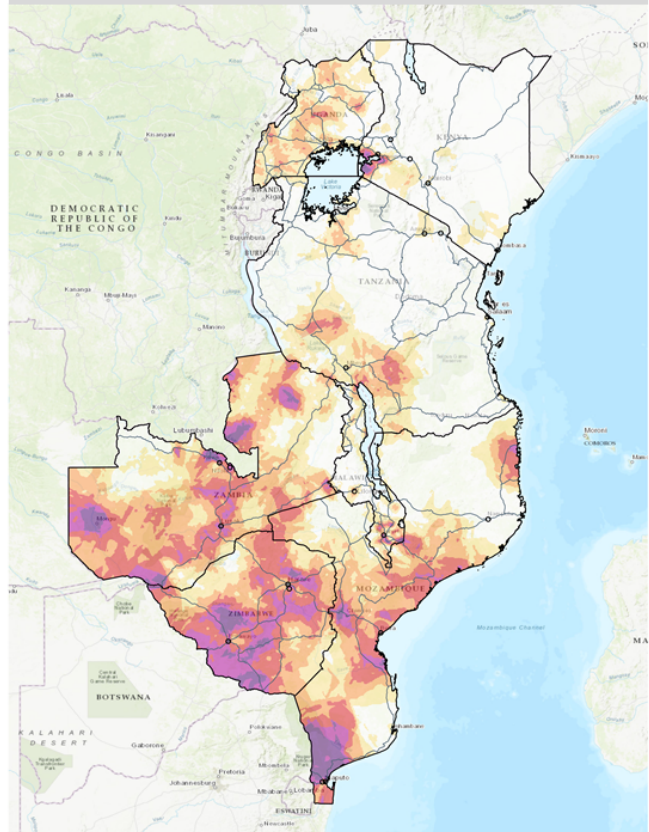

Supplement: S10 Fig — Maps and scatterplots illustrating the difference in HIV prevalence (per 5 km2 grid cell) between women and men (A and C, respectively) and between adults and young adults (B and D, respectively), for 7 countries of Eastern and Southern Africa. (PDF) [file pmed.1003042.s012.pdf]

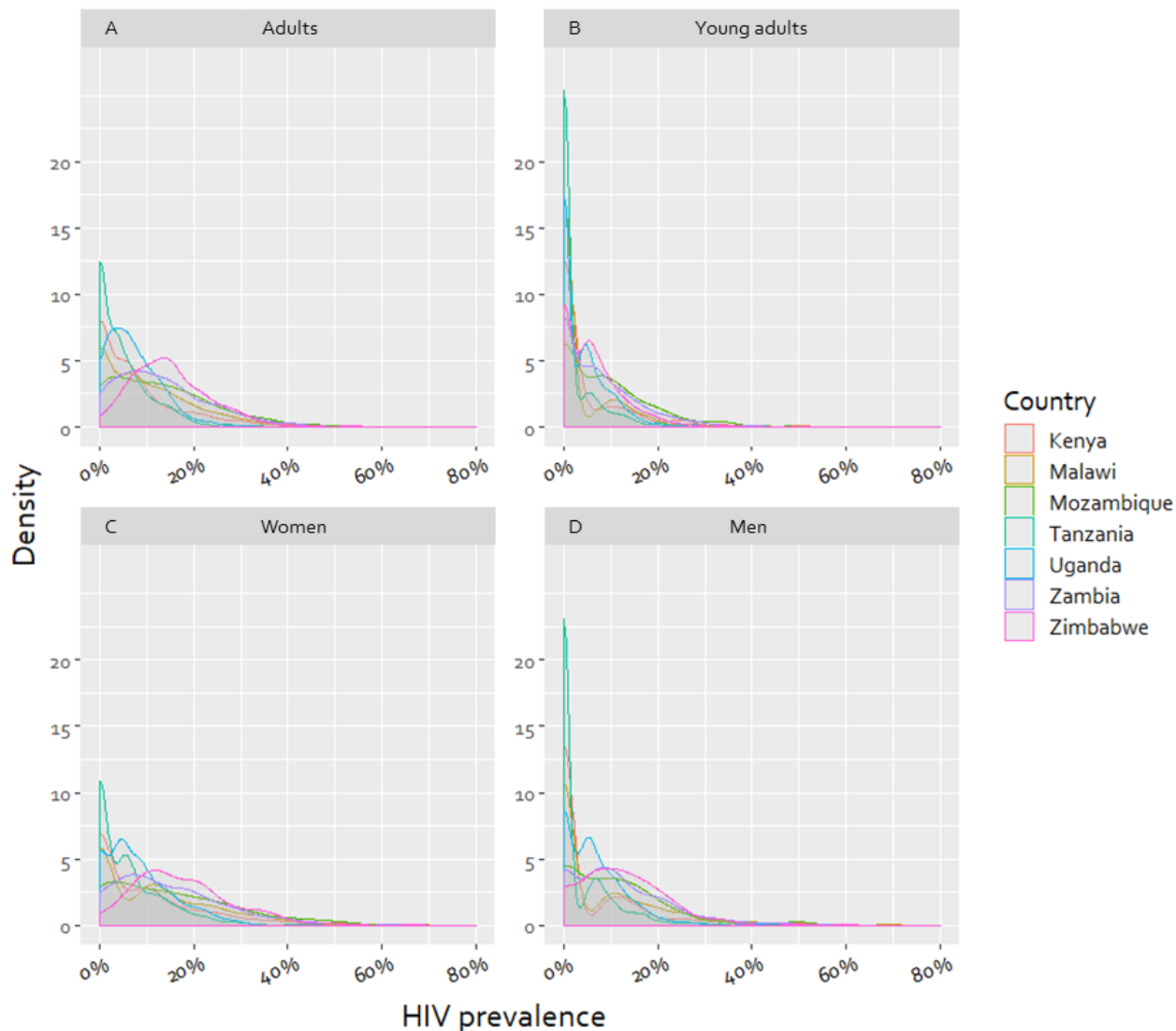

Supplement: S11 Fig — Density plots illustrating the overall sample location-level distributions of HIV prevalence among adults (A), young adults (B), women (C), and men (D) for each country included in this study. (PDF) [file pmed.1003042.s013.pdf]

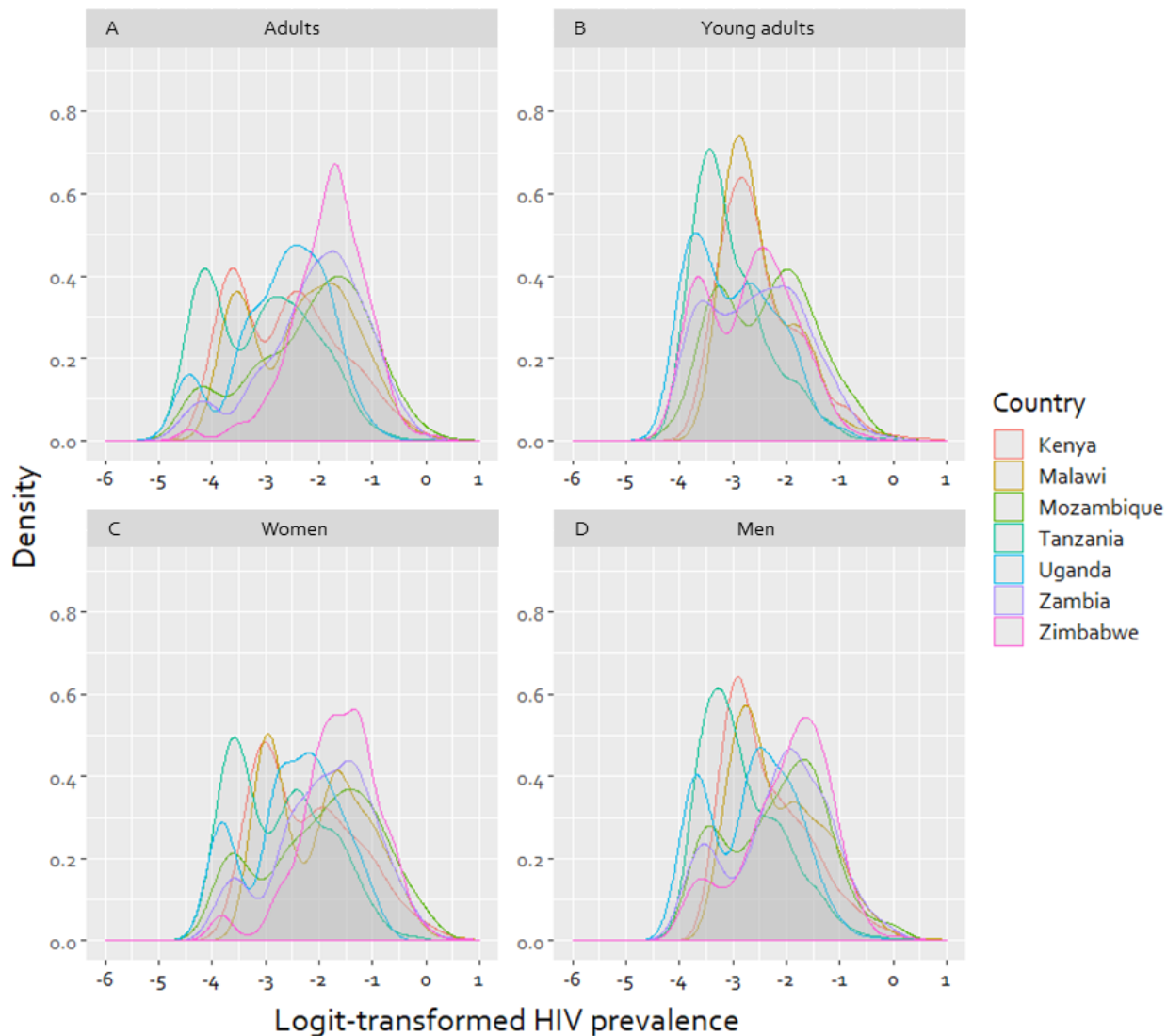

Supplement: S12 Fig — Density plots illustrating the overall logit-transformed sample location-level distributions of HIV prevalence among adults (A), young adults (B), women (C), and men (D) for each country included in this study, as used for semivariogram modelling and ordinary kriging. The logit-transformed HIV prevalence of −6 (on the x-axis) represents a prevalence of 0%, −5 of 1%, −4 of 2%, −3 of 5%, −2 of 12%, −1 of 27%, 0 of 50%, and 1 of 73. (PDF) [file pmed.1003042.s014.pdf]

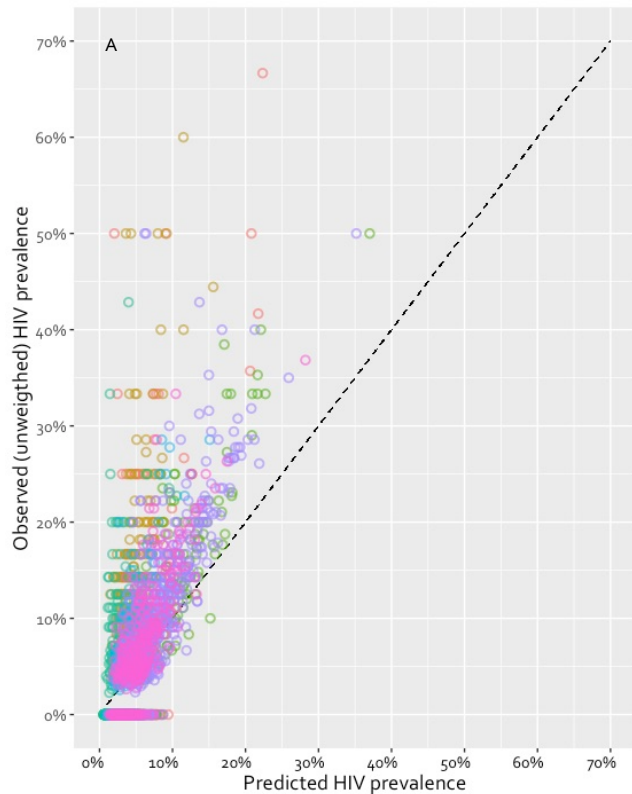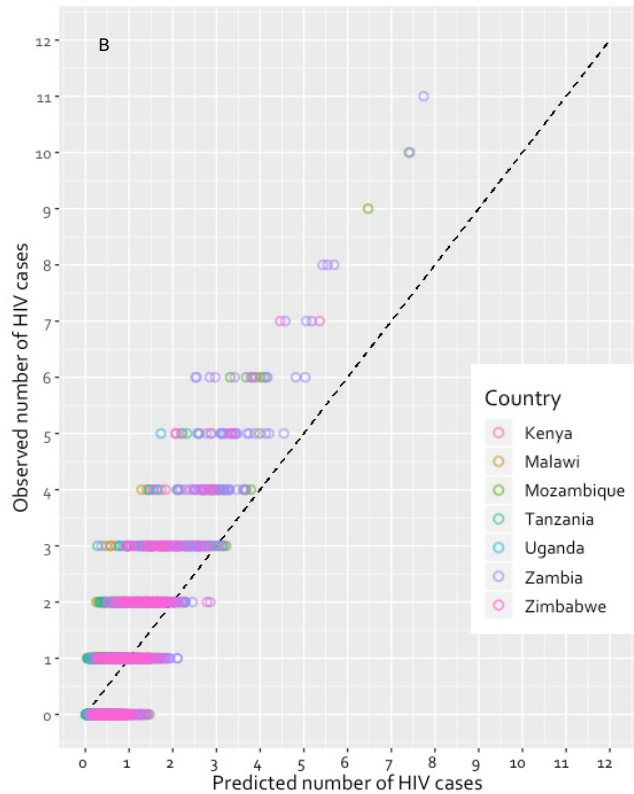

Supplement: S13 Fig — Plots illustrating the observed versus the predicted sample location-level HIV prevalence (A) and the observed versus the predicted number of HIV cases (B) among young adults (women 15–24 years and men 15–29 years) for the combined ‘full’ best-fitting multiple multilevel regression model per DHS sample location for 7 countries of Eastern and Southern Africa (also see S9 Table). (PDF) [file pmed.1003042.s015.pdf]
