# Supplementary material for: Mapping and characterising areas with high levels of HIV transmission in sub-Saharan Africa: A geospatial analysis of national survey data
Source: PLoS Med. 2020 Mar 6;17(3):e1003042. doi: 10.1371/journal.pmed.1003042 (PMC7059914; doi:10.1371/journal.pmed.1003042)
Supplement: S14 Fig — (PDF) [file pmed.1003042.s016.pdf]

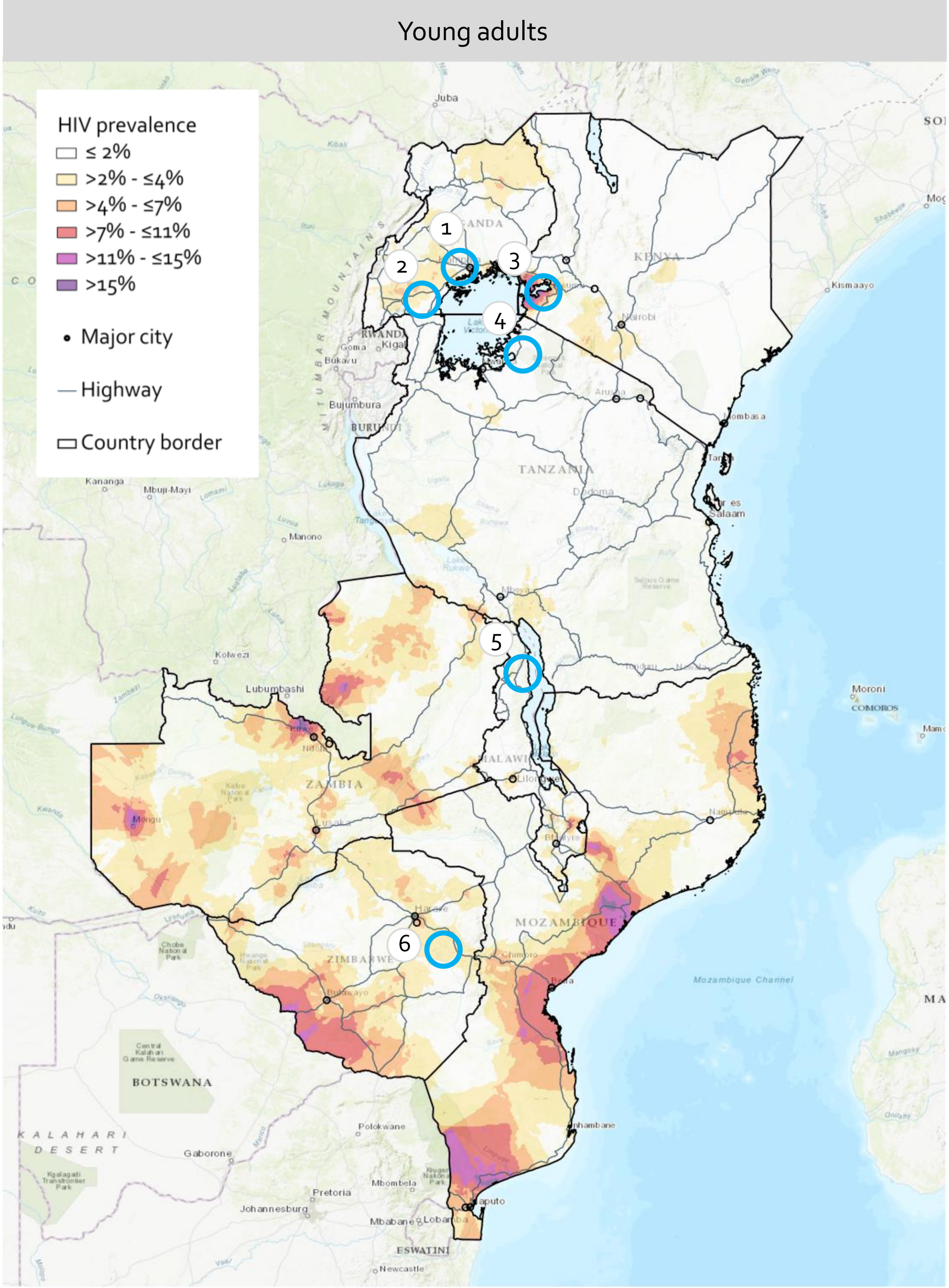

1 Masaka, Uganda
Prevalence young adults
ALPHA = ~2%
Our study = ≤2% to >2% - ≤ 4%

2 Rakai, Uganda
Prevalence young adults
ALPHA = ~10%
Our study = ≤2% to >4% - ≤ 7%

3 Kisumu, Kenya
Prevalence young adults
ALPHA = ~11%
Our study = >4%- ≤ 7% to >15%

4 Kisesa, Tanzania
Prevalence young adults
ALPHA = ~2%
Our study = ≤2%

5 Karonga, Malawi
Prevalence young adults
ALPHA = ~3%
Our study = ≤2%

6 Manicaland, Zimbabwe
Prevalence young adults
ALPHA = ~8%
Our study = >2% - ≤4%
