# Supplementary material for: Mapping and characterising areas with high levels of HIV transmission in sub-Saharan Africa: A geospatial analysis of national survey data
Source: PLoS Med. 2020 Mar 6;17(3):e1003042. doi: 10.1371/journal.pmed.1003042 (PMC7059914; doi:10.1371/journal.pmed.1003042)
Supplement: S1 Table — (DOCX) [file pmed.1003042.s017.docx]

**S1 Table. Overview of all variables included in the study.**

| **Variable name** | **Original value type** | **Scale or levels** | **Type of transformation for regression analysis** | **How were they measured?** | **Data source** | **Website** |
| --- | --- | --- | --- | --- | --- | --- |
| HIV status | Dichotomous | 0, 1 | N/A | HIV testing | DHSs, AISs | <https://dhsprogram.com/> |
| Sex | Dichotomous | 1, 2 | N/A | Self-reported | DHSs, AISs | <https://dhsprogram.com/> |
| Age | Continuous | 15 - 54 | Categorised; 5-year age groups | Self-reported | DHSs, AISs | <https://dhsprogram.com/> |
| Lifetime number of sex partners | Continuous | 0 - 95 | Categorised; 4 levels | Self-reported | DHSs, AISs | <https://dhsprogram.com/> |
| Number of sex partners past 12 months | Continuous | 0 - 95 | Categorised; 4 levels | Self-reported | DHSs, AISs | <https://dhsprogram.com/> |
| STI or signs of STI past 12 months | Dichotomous | 0, 1 | N/A | Self-reported | DHSs, AISs | <https://dhsprogram.com/> |
| Condom used last sexual intercourse | Dichotomous | 0, 1 | N/A | Self-reported | DHSs, AISs | <https://dhsprogram.com/> |
| Circumcised (only men) | Dichotomous | 0, 1 | N/A | Self-reported | DHSs, AISs | <https://dhsprogram.com/> |
| Paid for sexual intercourse 12 months (only men) | Dichotomous | 0, 1 | N/A | Self-reported | DHSs, AISs | <https://dhsprogram.com/> |
| Education | Categories | 4 levels;  0, 1, 2, 3 | N/A | Self-reported | DHSs, AISs | <https://dhsprogram.com/> |
| Wealth index | Categories | 5 levels;  1, 2, 3, 4, 5 | N/A | Weighted index | DHSs, AISs | <https://dhsprogram.com/> |
| Occupation | Categories | 11 levels;  1 – 10, 98 | N/A | Self-reported | DHSs, AISs | <https://dhsprogram.com/> |
| Type of resident | Dichotomous | 1, 2 | N/A | Self-reported | DHSs, AISs | <https://dhsprogram.com/> |
| Type of place of residence | Dichotomous | 1, 2 | N/A | Estimated by USAID | DHSs, AISs | <https://dhsprogram.com/> |
| Population density (per km^2^) | Continuous | 0 – 40,000 | Categorised; 6 levels | Estimated based on national census data | WorldPop (incorporated in DHSs) | <https://www.worldpop.org/> |
| Proximity to nearest highway (km) | Continuous | 0 - 431 | Categorised; 4 levels | Calculated (using ArcGIS Pro) | GDAM | <https://www.gadm.org/> |
| Proximity to nearest major city (km) | Continuous | 0 - 1531 | Categorised; 5 levels | Calculated (using ArcGIS Pro) | World Population Review | [www.worldpopulationreview.com/worldcities/](http://www.worldpopulationreview.com/worldcities/) |
| Proximity to nearest border crossing or port (km) | Continuous | 0 - 523 | Categorised; 5 levels | Calculated (using ArcGIS Pro) | Southern Africa Integrated Regional Transport Program report (2010) | N/A |
| Enhanced vegetation index (EVI) | Continuous | 0 - 234 | Categorised; 6 levels | Satellite imaging | NASA, (incorporated in DHSs) | <http://sedac.ciesin.columbia.edu/> |
| Global human footprint (GHF) (%) | Continuous | 5 - 100 | Categorised; 5 levels | Estimated by NASA, using several data sources | NASA, (incorporated in DHSs) | <http://sedac.ciesin.columbia.edu/> |

AISs = AIDS Indicator Surveys, DHSs = Demographic and Health Surveys, NASA = National Aeronautics and Space Administration, USAID = United States Agency for International Development
