# Supplementary material for: Mapping and characterising areas with high levels of HIV transmission in sub-Saharan Africa: A geospatial analysis of national survey data
Source: PLoS Med. 2020 Mar 6;17(3):e1003042. doi: 10.1371/journal.pmed.1003042 (PMC7059914; doi:10.1371/journal.pmed.1003042)
Supplement: S2 Table — (DOCX) [file pmed.1003042.s018.docx]

**S2 Table. Spatial autocorrelation of HIV prevalence at the sample location-level, estimated by Moran’s *I* index.**

|  | **Observed value** | **Standard deviation** | | | **p-value** | |
| --- | --- | --- | --- | --- | --- | --- |
|  |  | |  |  | |  |
| **Adults** |  | |  |  | |  |
| *Countries overall* | *0.13* | | *0.002* | *<0.001* | | ***** |
| Kenya | 0.11 | | 0.009 | <0.001 | | *** |
| Malawi | 0.10 | | 0.005 | <0.001 | | *** |
| Mozambique | 0.24 | | 0.016 | <0.001 | | *** |
| Tanzania | 0.12 | | 0.007 | <0.001 | | *** |
| Uganda | 0.06 | | 0.007 | <0.001 | | *** |
| Zambia | 0.12 | | 0.008 | <0.001 | | *** |
| Zimbabwe | 0.02 | | 0.012 | 0.024 | | * |
|  |  | |  |  | |  |
| **Young adults** |  | |  |  | |  |
| *Countries overall* | *0.05* | | *0.002* | *<0.001* | | ***** |
| Kenya | 0.05 | | 0.009 | <0.001 | | *** |
| Malawi | 0.02 | | 0.005 | <0.001 | | *** |
| Mozambique | 0.05 | | 0.016 | <0.001 | | *** |
| Tanzania | 0.03 | | 0.007 | <0.001 | | *** |
| Uganda | 0.01 | | 0.007 | <0.001 | | *** |
| Zambia | 0.05 | | 0.008 | <0.001 | | *** |
| Zimbabwe | 0.02 | | 0.012 | 0.067 | | . |
|  |  | |  |  | |  |
| **Women** |  | |  |  | |  |
| *Countries overall* | *0.11* | | *0.002* | *<0.001* | | ***** |
| Kenya | 0.09 | | 0.009 | <0.001 | | *** |
| Malawi | 0.09 | | 0.005 | <0.001 | | *** |
| Mozambique | 0.22 | | 0.016 | <0.001 | | *** |
| Tanzania | 0.11 | | 0.007 | <0.001 | | *** |
| Uganda | 0.08 | | 0.007 | <0.001 | | *** |
| Zambia | 0.12 | | 0.008 | <0.001 | | *** |
| Zimbabwe | 0.03 | | 0.012 | 0.002 | | ** |
|  |  | |  |  | |  |
| **Men** |  | |  |  | |  |
| *Countries overall* | *0.08* | | *0.002* | *<0.001* | | ***** |
| Kenya | 0.06 | | 0.009 | <0.001 | | *** |
| Malawi | 0.05 | | 0.005 | <0.001 | | *** |
| Mozambique | 0.12 | | 0.016 | <0.001 | | *** |
| Tanzania | 0.06 | | 0.007 | <0.001 | | *** |
| Uganda | 0.01 | | 0.007 | 0.140 | |  |
| Zambia | 0.06 | | 0.008 | <0.001 | | *** |
| Zimbabwe | 0.02 | | 0.012 | 0.077 | | . |
|  |  | |  |  | |  |

Significance codes: 0 ‘***’ 0.001 ‘**’ 0.01 ‘*’ 0.05 ‘.’ 0.1 ‘ ’ 1
