# Supplementary material for: Mapping and characterising areas with high levels of HIV transmission in sub-Saharan Africa: A geospatial analysis of national survey data
Source: PLoS Med. 2020 Mar 6;17(3):e1003042. doi: 10.1371/journal.pmed.1003042 (PMC7059914; doi:10.1371/journal.pmed.1003042)
Supplement: S3 Table — Data obtained through (https://dhsprogram.com/). (DOCX) [file pmed.1003042.s019.docx]

**S3 Table. Bivariate logistic regression models of HIV status and behavioural, socioeconomic and environmental variables in young adults (women 15-24 years and men 15-29 years of age) in seven countries of Eastern and Southern Africa, adjusted for age and sex.** Total number of observations (*N*) is 53,234, total number of DHS sample locations is 3,665, overall HIV prevalence is 4.9%. Data obtained through (<https://dhsprogram.com/>).

|  | **Young adults** | | | | |
| --- | --- | --- | --- | --- | --- |
| **Covariate** | ***N*** | **HIV prevalence (%)** | **aOR [95% CI]** | **p-value** | |
| **Lifetime number of sex partners** | | | | | |
| None | 16,532 | 2.4 | 1 |  |  |
| 1-3 | 27,739 | 5.6 | 1.60 [1.48; 1.73] | <0.001 | *** |
| 4-9 | 7,002 | 7.3 | 2.53 [2.37; 2.69] | <0.001 | *** |
| 10+ | 1,961 | 8.5 | 3.18 [2.96; 3.39] | <0.001 | *** |
| Sex |  |  |  |  |  |
| Male | 27,698 | 4.0 | 1 |  |  |
| Female | 25,536 | 6.0 | 2.33 [2.23; 2.44] | <0.001 | *** |
| Age (per 5-year age group) |  |  |  |  |  |
| 15-19 | 25,586 | 3.0 | 1 |  |  |
| 20-24 | 20,548 | 6.7 | 1.82 [1.71; 1.92] | <0.001 | *** |
| 25-29 | 7,100 | 7.0 | 2.71 [2.55; 2.86] | <0.001 | *** |
|  |  |  |  |  |  |
| **Number of sex partners past 12 months** | | | | | |
| None | 21,802 | 3.2 | 1 |  |  |
| 1 | 27,206 | 6.1 | 1.35 [1.24; 1.45] | <0.001 | *** |
| 2-3 | 3,902 | 6.5 | 1.67 [1.50; 1.84] | <0.001 | *** |
| 4+ | 324 | 6.5 | 1.77 [1.29; 2.25] | 0.019 | * |
| Sex |  |  |  |  |  |
| Male | 27,698 | 4.0 | 1 |  |  |
| Female | 25,536 | 6.0 | 2.09 [1.99; 2.19] | <0.001 | *** |
| Age (per 5-year age group) |  |  |  |  |  |
| 15-19 | 25,586 | 3.0 | 1 |  |  |
| 20-24 | 20,548 | 6.7 | 2.05 [1.94; 2.15] | <0.001 | *** |
| 25-29 | 7,100 | 7.0 | 3.30 [3.15; 3.45] | <0.001 | *** |
|  |  |  |  |  |  |
| **STI or signs of STI past 12 months** | | | | | |
| No | 49,351 | 4.6 | 1 |  |  |
| Yes | 3,883 | 9.1 | 1.82 [1.69; 1.95] | <0.001 | *** |
| Sex |  |  |  |  |  |
| Male | 27,698 | 4.0 | 1 |  |  |
| Female | 25,536 | 6.0 | 2.13 [2.03; 3.92] | <0.001 | *** |
| Age (per 5-year age group) |  |  |  |  |  |
| 15-19 | 25,586 | 3.0 | 1 |  |  |
| 20-24 | 20,548 | 6.7 | 2.22 [2.12; 2.31] | <0.001 | *** |
| 25-29 | 7,100 | 7.0 | 3.78 [3646; 3.92] | <0.001 | *** |
|  |  |  |  |  |  |
| **Condom used last sexual intercourse** | | | | | |
| No | 44,167 | 4.7 | 1 |  |  |
| Yes | 9,067 | 6.2 | 1.35 [1.24; 1.45] | <0.001 | *** |
| Sex |  |  |  |  |  |
| Male | 27,698 | 4.0 | 1 |  |  |
| Female | 25,536 | 6.0 | 2.17 [2.07; 2.27] | <0.001 | *** |
| Age (per 5-year age group) |  |  |  |  |  |
| 15-19 | 25,586 | 3.0 | 1 |  |  |
| 20-24 | 20,548 | 6.7 | 2.27 [2.17; 2.36] | <0.001 | *** |
| 25-29 | 7,100 | 7.0 | 3.96 [3.82; 4.10] | <0.001 | *** |
|  |  |  |  |  |  |
| **Circumcised (only men)** | | | | | |
| No | 17,250 | 4.6 | 1 |  |  |
| Yes | 10,448 | 2.9 | 0.65 [0.51; 0.79] | <0.001 | *** |
| Sex |  |  |  |  |  |
| Male | 27,698 | 4.0 | 1 |  |  |
| Female | 25,536 | 6.0 | 2.19 [2.08; 2.29] | <0.001 | *** |
| Age (per 5-year age group) |  |  |  |  |  |
| 15-19 | 25,586 | 3.0 | 1 |  |  |
| 20-24 | 20,548 | 6.7 | 2.32 [2.23; 2.41] | <0.001 | *** |
| 25-29 | 7,100 | 7.0 | 3.95 [3.81; 4.09] | <0.001 | *** |
|  |  |  |  |  |  |
|  | | | | | |

**[Continued] S2 Table. Univariate logistic regression models of HIV status and behavioural, socioeconomic and environmental variables in young adults (women 15-24 years and men 15-29 years of age) in seven countries of Eastern and Southern Africa, adjusted for age and sex.** Total number of observations (*N*) is 53,234, total number of DHS sample locations is 3,665, overall HIV prevalence is 4.9%. Data obtained through (<https://dhsprogram.com/>).

|  | **Young adults** | | | | |
| --- | --- | --- | --- | --- | --- |
| **Covariate** | ***N*** | **HIV prevalence (%)** | **aOR [95% CI]** | **p-value** |  |
| **Paid for sexual intercourse 12 months (only men)** | | | | | |
| No | 25,871 | 3.9 | 1 |  |  |
| Yes | 1,827 | 5.3 | 1.33 [1.11; 1.55] | 0.012 | * |
| Sex |  |  |  |  |  |
| Male | 27,698 | 4.0 | 1 |  |  |
| Female | 25,536 | 6.0 | N/A |  |  |
| Age (per 5-year age group) |  |  |  |  |  |
| 15-19 | 25,586 | 3.0 | 1 |  |  |
| 20-24 | 20,548 | 6.7 | 2.31 [2.21; 2.40] | <0.001 | *** |
| 25-29 | 7,100 | 7.0 | 3.97 [3.83; 4.11] | <0.001 | *** |
|  |  |  |  |  |  |
| **Education** |  |  |  |  |  |
| No education | 2,410 | 5.2 | 0.92 [0.71; 1.12] | 0.406 |  |
| Primary | 25,013 | 4.8 | 1 |  |  |
| Secondary | 23,956 | 5.1 | 0.93 [0.84; 1.03] | 0.150 |  |
| Higher | 1,855 | 4.5 | 0.62 [0.38; 0.86] | <0.001 | *** |
| Sex |  |  |  |  |  |
| Male | 27,698 | 4.0 | 1 |  |  |
| Female | 25,536 | 6.0 | 2.07 [1.97; 2.17] | <0.001 | *** |
| Age (per 5-year age group) |  |  |  |  |  |
| 15-19 | 25,586 | 3.0 | 1 |  |  |
| 20-24 | 20,548 | 6.7 | 2.36 [2.27; 2.46] | <0.001 | *** |
| 25-29 | 7,100 | 7.0 | 4.10 [3.96; 4.24] | <0.001 | *** |
|  |  |  |  |  |  |
| **Wealth index** |  |  |  |  |  |
| 1 ‘poorest’ | 8,102 | 3.8 | 1 |  |  |
| 2 | 9,307 | 3.8 | 1.00 [0.84; 1.16] | 0.998 |  |
| 3 | 10,027 | 4.8 | 1.26 [1.10; 1.42] | 0.004 | ** |
| 4 | 11,691 | 5.9 | 1.44 [1.29; 1.60] | <0.001 | *** |
| 5 ‘wealthiest’ | 14,107 | 5.6 | 1.36 [1.20; 1.51] | <0.001 | *** |
| Sex |  |  |  |  |  |
| Male | 27,698 | 4.0 | 1 |  |  |
| Female | 25,536 | 6.0 | 2.09 [1.99; 2.18] | <0.001 | *** |
| Age (per 5-year age group) |  |  |  |  |  |
| 15-19 | 25,586 | 3.0 | 1 |  |  |
| 20-24 | 20,548 | 6.7 | 2.31 [2.22; 2.41] | <0.001 | *** |
| 25-29 | 7,100 | 7.0 | 3.98 [3.84; 4.12] | <0.001 | *** |
|  |  |  |  |  |  |
| **Occupation** |  |  |  |  |  |
| Not working | 21,467 | 4.5 | 1 |  |  |
| Professional/technical/managerial | 1,857 | 5.8 | 0.97 [0.74; 1.19] | 0.758 |  |
| Clerical | 310 | 6.8 | 1.06 [0.58; 1.53] | 0.815 |  |
| Sales | 3,619 | 9.0 | 1.43 [1.29; 1.58] | <0.001 | *** |
| Agricultural – self employed | 7,975 | 4.4 | 0.92 [0.77; 1.06] | 0.235 |  |
| Agricultural – employee | 7,055 | 3.2 | 0.73 [0.56; 0.89] | <0.001 | *** |
| Household/domestic | 1,053 | 6.3 | 1.32 [1.05; 1.59] | 0.046 | * |
| Services | 2,154 | 7.5 | 1.43 [1.24; 1.62] | <0.001 | *** |
| Skilled manual | 3,474 | 6.1 | 1.21 [1.04; 1.39] | 0.030 | * |
| Unskilled manual | 4,008 | 4.6 | 0.94 [0.76; 1.12] | 0.220 |  |
| Don’t know | 262 | 3.1 | 0.78 [0.04; 1.52] | 0.508 |  |
| Sex |  |  |  |  |  |
| Male | 27,698 | 4.0 | 1 |  |  |
| Female | 25,536 | 6.0 | 2.06 [1.96; 2.16] | <0.001 | *** |
| Age (per 5-year age group) |  |  |  |  |  |
| 15-19 | 25,586 | 3.0 | 1 |  |  |
| 20-24 | 20,548 | 6.7 | 2.24 [2.15; 2.34] | <0.001 | *** |
| 25-29 | 7,100 | 7.0 | 3.78 [3.63; 3.93] | <0.001 | *** |
|  |  |  |  |  |  |

**[Continued] S2 Table. Univariate logistic regression models of HIV status and behavioural, socioeconomic and environmental variables in young adults (women 15-24 years and men 15-29 years of age) in seven countries of Eastern and Southern Africa, adjusted for age and sex.** Total number of observations (*N*) is 53,234, total number of DHS sample locations is 3,665, overall HIV prevalence is 4.9%. Data obtained through (<https://dhsprogram.com/>).

|  | **Young adults** | | | | |
| --- | --- | --- | --- | --- | --- |
| **Covariate** | ***N*** | **HIV prevalence (%)** | **aOR [95% CI]** | **p-value** |  |
| **Type of resident** |  |  |  |  |  |
| ‘De jure’ (usual resident) | 51,616 | 4.9 | 1 |  |  |
| Only ‘de facto’ (slept in the house last night) | 1,618 | 5.5 | 1.11 [0.88; 1.34] | 0.376 |  |
| Sex |  |  |  |  |  |
| Male | 27,698 | 4.0 | 1 |  |  |
| Female | 25,536 | 6.0 | 2.08 [1.98; 2.18] | <0.001 | *** |
| Age (per 5-year age group) |  |  |  |  |  |
| 15-19 | 25,586 | 3.0 | 1 |  |  |
| 20-24 | 20,548 | 6.7 | 2.31 [2.22; 2.40] | <0.001 | *** |
| 25-29 | 7,100 | 7.0 | 3.96 [3.83; 4.10] | <0.001 | *** |
|  |  |  |  |  |  |
| **Type of place of residence** |  |  |  |  |  |
| Urban | 18,519 | 7.2 | 1 |  |  |
| Rural | 34,715 | 3.7 | 0.49 [0.39; 0.60] | <0.001 | *** |
| Sex |  |  |  |  |  |
| Male | 27,698 | 4.0 | 1 |  |  |
| Female | 25,536 | 6.0 | 2.07 [1.97; 2.17] | <0.001 | *** |
| Age (per 5-year age group) |  |  |  |  |  |
| 15-19 | 25,586 | 3.0 | 1 |  |  |
| 20-24 | 20,548 | 6.7 | 2.28 [2.18; 2.37] | <0.001 | *** |
| 25-29 | 7,100 | 7.0 | 3.90 [3.76; 4.04] | <0.001 | *** |
|  |  |  |  |  |  |
| **Population density (per km^2^)** |  |  |  |  |  |
| ≤25 | 12,673 | 5.3 | 1.35 [1.17; 1.53] | <0.001 | *** |
| >25 - ≤50 | 6,996 | 4.2 | 1.06 [0.85; 1.27] | 0.575 |  |
| >50 - ≤100 | 4,650 | 3.2 | 0.76 [0.51; 1.01] | 0.033 | * |
| >100 - ≤250 | 8,182 | 4.0 | 1 |  |  |
| >250 - ≤500 | 6,602 | 4.4 | 1.08 [0.87; 1.30] | 0.457 |  |
| >500 | 14,131 | 6.4 | 1.66 [1.49; 1.84] | <0.001 | *** |
| Sex |  |  |  |  |  |
| Male | 27,698 | 4.0 | 1 |  |  |
| Female | 25,536 | 6.0 | 2.08 [1.98; 2.18] | <0.001 | *** |
| Age (per 5-year age group) |  |  |  |  |  |
| 15-19 | 25,586 | 3.0 | 1 |  |  |
| 20-24 | 20,548 | 6.7 | 2.30 [2.20; 2.39] | <0.001 | *** |
| 25-29 | 7,100 | 7.0 | 3.92 [3.78; 4.06] | <0.001 | *** |
|  |  |  |  |  |  |
| **Proximity to nearest highway (km)** |  |  |  |  |  |
| ≤5 | 19,404 | 5.8 | 1 |  |  |
| >5 - ≤10 | 5,670 | 4.7 | 0.79 [0.60; 0.97] | 0.012 | ** |
| >10 - ≤50 | 17,168 | 3.9 | 0.67 [0.54; 0.80] | <0.001 | *** |
| >50 | 10,992 | 5.1 | 0.87 [0.72; 1.01] | <0.001 | *** |
| Sex |  |  |  |  |  |
| Male | 27,698 | 4.0 | 1 |  |  |
| Female | 25,536 | 6.0 | 2.08 [1.98; 2.18] | <0.001 | *** |
| Age (per 5-year age group) |  |  |  |  |  |
| 15-19 | 25,586 | 3.0 | 1 |  |  |
| 20-24 | 20,548 | 6.7 | 2.30 [2.21; 2.40] | <0.001 | *** |
| 25-29 | 7,100 | 7.0 | 3.94 [3.80; 4.08] | <0.001 | *** |
|  |  |  |  |  |  |
| **Proximity to nearest major city (km)** |  |  |  |  |  |
| ≤10 | 6,114 | 5.9 | 1.30 [1.13; 1.47] | 0.003 | ** |
| >10 - ≤50 | 6,221 | 4.5 | 0.95 [0.78; 1.13] | 0.572 |  |
| >50 - ≤100 | 7,986 | 5.1 | 1.09 [0.94; 1.24] | 0.262 |  |
| >100 - ≤500 | 31,879 | 4.7 | 1 |  |  |
| >500 | 1,034 | 9.4 | 2.20 [1.88; 2.53] | <0.001 | *** |
| Sex |  |  |  |  |  |
| Male | 27,698 | 4.0 | 1 |  |  |
| Female | 25,536 | 6.0 | 2.08 [1.98; 2.18] | <0.001 | *** |
| Age (per 5-year age group) |  |  |  |  |  |
| 15-19 | 25,586 | 3.0 | 1 |  |  |
| 20-24 | 20,548 | 6.7 | 2.31 [2.21; 2.40] | <0.001 | *** |
| 25-29 | 7,100 | 7.0 | 3.95 [3.81; 4.09] | <0.001 | *** |
|  |  |  |  |  |  |

**[Continued] S2 Table. Univariate logistic regression models of HIV status and behavioural, socioeconomic and environmental variables in young adults (women 15-24 years and men 15-29 years of age) in seven countries of Eastern and Southern Africa, adjusted for age and sex.** Total number of observations (*N*) is 53,234, total number of DHS sample locations is 3,665, overall HIV prevalence is 4.9%. Data obtained through (<https://dhsprogram.com/>).

|  | **Young adults** | | | | |
| --- | --- | --- | --- | --- | --- |
| **Covariate** | ***N*** | **HIV prevalence (%)** | **aOR [95% CI]** | **p-value** |  |
| **Proximity to nearest border crossing or port (km)** | |  |  |  |  |
| ≤10 | 2,103 | 6.6 | 1.32 [1.05; 1.59] | 0.041 | * |
| >10 - ≤50 | 5,366 | 4.8 | 0.93 [0.75; 1.12] | 0.469 |  |
| >50 - ≤100 | 9,225 | 3.9 | 0.79 [0.64; 0.94] | 0.003 | ** |
| >100 - ≤500 | 36,224 | 5.1 | 1 |  |  |
| >500 | 316 | 9.8 | 2.00 [1.37; 2.62] | 0.030 | * |
| Sex |  |  |  |  |  |
| Male | 27,698 | 4.0 | 1 |  |  |
| Female | 25,536 | 6.0 | 2.08 [1.98; 2.18] | <0.001 | *** |
| Age (per 5-year age group) |  |  |  |  |  |
| 15-19 | 25,586 | 3.0 | 1 |  |  |
| 20-24 | 20,548 | 6.7 | 2.31 [2.21; 2.09] | <0.001 | *** |
| 25-29 | 7,100 | 7.0 | 3.96 [3.82; 4.09] | <0.001 | *** |
|  |  |  |  |  |  |
| **Enhanced vegetation index (EVI)** |  |  |  |  |  |
| ≤51 (water bodies, no DHS clusters) | N/A | N/A | N/A | N/A |  |
| >51 - ≤76 | 495 | 4.1 | 0.59 [-0.04; 1.22] | 0.105 |  |
| >76 - ≤102 | 1,724 | 4.1 | 0.73 [0.40; 1.05] | 0.051 | . |
| >102 - ≤137 | 9,405 | 5.1 | 0.97 [0.82;1.13] | 0.715 |  |
| >137 - ≤181 | 22,949 | 5.0 | 1 | 0.718 |  |
| >181 - ≤250 | 18,661 | 4.9 |  |  |  |
| Sex |  |  |  |  |  |
| Male | 27,698 | 4.0 | 1 |  |  |
| Female | 25,536 | 6.0 | 2.08 [1.98; 2.18] | <0.001 | *** |
| Age (per 5-year age group) |  |  |  |  |  |
| 15-19 | 25,586 | 3.0 | 1 |  |  |
| 20-24 | 20,548 | 6.7 | 2.32 [2.22; 2.41] | <0.001 | *** |
| 25-29 | 7,100 | 7.0 | 3.97 [3.83; 4.101] | <0.001 | *** |
|  |  |  |  |  |  |
| **Global human footprint (GHF) (%)** |  |  |  |  |  |
| ≤17 | 2,028 | 4.3 | 1.14 [0.84; 1.43] | 0.393 |  |
| >17 - ≤29 | 15,924 | 3.8 | 1 |  |  |
| >29 - ≤41 | 18,675 | 4.3 | 1.15 [1.01; 1.29] | 0.048 | * |
| >41 - ≤57 | 6,359 | 6.7 | 1.82 [1.64; 2.00] | <0.001 | *** |
| >57 - ≤100 | 10,248 | 6.9 | 1.92 [1.76; 2.07] | <0.001 | *** |
| Sex |  |  |  |  |  |
| Male | 27,698 | 4.0 | 1 |  |  |
| Female | 25,536 | 6.0 | 2.08 [1.98; 2.18] | <0.001 | *** |
| Age (per 5-year age group) |  |  |  |  |  |
| 15-19 | 25,586 | 3.0 | 1 |  |  |
| 20-24 | 20,548 | 6.7 | 2.28 [2.19; 2.38] | <0.001 | *** |
| 25-29 | 7,100 | 7.0 | 3.93 [3.79; 4.07] | <0.001 | *** |
|  |  |  |  |  |  |

Significance codes: 0 ‘***’ 0.001 ‘**’ 0.01 ‘*’ 0.05 ‘.’ 0.1 ‘ ’ 1
