# Supplementary material for: Mapping and characterising areas with high levels of HIV transmission in sub-Saharan Africa: A geospatial analysis of national survey data
Source: PLoS Med. 2020 Mar 6;17(3):e1003042. doi: 10.1371/journal.pmed.1003042 (PMC7059914; doi:10.1371/journal.pmed.1003042)
Supplement: S4 Table — Data obtained through (https://dhsprogram.com/). (DOCX) [file pmed.1003042.s020.docx]

**S4 Table. Multiple multilevel nested ‘empty’ logistic regression model of HIV status in young adults (women 15-24 years and men 15-29 years of age) for seven countries of Eastern and Southern Africa, adjusted for age and sex.** Data obtained through (<https://dhsprogram.com/>).

|  | **Young adults** | | | | |
| --- | --- | --- | --- | --- | --- |
| **Covariate** | ***N*** | **HIV prevalence (%)** | **aOR [95% CI]** | **p-value** | |
| Sex | | | | | |
| Male | 27,698 | 4.0 | 1 |  |  |
| Female | 25,536 | 6.0 | 2.08 [1.98; 2.18] | <0.001 | *** |
| Age (per 5-year age group) | | | | | |
| 15-19 | 25,586 | 3.0 | 1 |  |  |
| 20-24 | 20,548 | 6.7 | 2.31 [2.22; 2.41] | <0.001 | *** |
| 25-29 | 7,100 | 7.0 | 3.97 [3.83; 4.10] | <0.001 | *** |
|  |  |  |  |  |  |
| *Model summary: AIC = 19,822.9; BIC = 19,867.3; logLik = -9,906.4; DF = 53,229; Deviance = 19,812.9*  *Random effect (CLUST.ID): Variance = 0.852; SD = 0.923* | | | | | |

Significance codes: 0 ‘***’ 0.001 ‘**’ 0.01 ‘*’ 0.05 ‘.’ 0.1 ‘ ’ 1

*N* = Number of observations, aOR = Adjusted Odds Ratio, CI = Confidence Interval, AIC = Akaike Information Criterion, BIC = Bayesian Information Criterion, logLik = log likelihood, DF = Degrees of Freedom, SD = Standard Deviation, N/A = Not Applicable, ‘-’ = Covariate not present in regression model
