# Supplementary material for: Mapping and characterising areas with high levels of HIV transmission in sub-Saharan Africa: A geospatial analysis of national survey data
Source: PLoS Med. 2020 Mar 6;17(3):e1003042. doi: 10.1371/journal.pmed.1003042 (PMC7059914; doi:10.1371/journal.pmed.1003042)
Supplement: S5 Table — Data obtained through (https://dhsprogram.com/). (DOCX) [file pmed.1003042.s021.docx]

**S5 Table. Multiple multilevel logistic regression model of HIV status and behavioural variables in young adults (women 15-24 years and men 15-29 years of age) for seven countries of Eastern and Southern Africa, adjusted for age and sex.** Data obtained through (<https://dhsprogram.com/>).

|  | **Young adults** | | | | |
| --- | --- | --- | --- | --- | --- |
| **Covariate** | ***N*** | **HIV prevalence (%)** | **aOR [95% CI]** | **p-value** | |
| **Lifetime number of sex partners** |  |  |  |  |  |
| None | 16,532 | 2.4 | 1 |  |  |
| 1-3 | 27,739 | 5.6 | 1.45 [1.32; 1.58] | <0.001 | *** |
| 4-9 | 7,002 | 7.3 | 2.20 [2.03; 2.37] | <0.001 | *** |
| 10+ | 1,961 | 8.5 | 2.74 [2.51; 2.96] | <0.001 | *** |
| **STI or signs of STI past 12 months** |  |  |  |  |  |
| No | 49,351 | 4.6 | 1 |  |  |
| Yes | 3,883 | 9.1 | 1.57 [1.44; 1.70] | <0.001 | *** |
| **Condom used last sexual intercourse** |  |  |  |  |  |
| No | 44,167 | 4.7 | 1 |  |  |
| Yes | 9,067 | 6.2 | 1.17 [1.07; 1.28] | 0.004 | ** |
| **Circumcised (only men)** |  |  |  |  |  |
| No | 17,250 | 4.6 | 1 |  |  |
| Yes | 10,448 | 2.9 | 0.62 [0.47; 0.76] | <0.001 | *** |
| Sex | | | | | |
| Male | 27,698 | 4.0 | 1 |  |  |
| Female | 25,536 | 6.0 | 2.03 [1.91; 2.12] | <0.001 | *** |
| Age (per 5-year age group) | | | | | |
| 15-19 | 25,586 | 3.0 | 1 |  |  |
| 20-24 | 20,548 | 6.7 | 1.81 [1.70; 1.91] | <0.001 | *** |
| 25-29 | 7,100 | 7.0 | 2.72 [2.57; 2.88] | <0.001 | *** |
|  |  |  |  |  |  |
| *Model summary: AIC = 19,579.0; BIC = 19,676.4; logLik = -9,778.5; DF = 53,223; Deviance = 19,557.0*  *Random effect (CLUST.ID): Variance = 0.801; SD = 0.895* | | | | | |
|  | | | | | |

Significance codes: 0 ‘***’ 0.001 ‘**’ 0.01 ‘*’ 0.05 ‘.’ 0.1 ‘ ’ 1

*N* = Number of observations, aOR = Adjusted Odds Ratio, CI = Confidence Interval, AIC = Akaike Information Criterion, BIC = Bayesian Information Criterion, logLik = log likelihood, DF = Degrees of Freedom, SD = Standard Deviation, N/A = Not Applicable, ‘-’ = Covariate not present in regression model
