# Supplementary material for: Mapping and characterising areas with high levels of HIV transmission in sub-Saharan Africa: A geospatial analysis of national survey data
Source: PLoS Med. 2020 Mar 6;17(3):e1003042. doi: 10.1371/journal.pmed.1003042 (PMC7059914; doi:10.1371/journal.pmed.1003042)
Supplement: S6 Table — Data obtained through (https://dhsprogram.com/). (DOCX) [file pmed.1003042.s022.docx]

**S6 Table. Multiple multilevel logistic regression model of HIV status and socioeconomic variables in young adults (women 15-24 years and men 15-29 years of age) for seven countries of Eastern and Southern Africa, adjusted for age and sex.** Data obtained through (<https://dhsprogram.com/>).

|  | **Young adults** | | | | |
| --- | --- | --- | --- | --- | --- |
| **Covariate** | ***N*** | **HIV prevalence (%)** | **aOR [95% CI]** | **p-value** | |
| **Education** |  |  |  |  |  |
| No education | 2,410 | 5.2 | 1.00 [0.80; 1.20] | 0.997 |  |
| Primary | 25,013 | 4.8 | 1 |  |  |
| Secondary | 23,956 | 5.1 | 0.83 [0.73; 0.93] | <0.001 | *** |
| Higher | 1,855 | 4.5 | 0.52 [0.26; 0.78] | <0.001 | *** |
| **Wealth index** |  |  |  |  |  |
| 1 ‘poorest’ | 8,102 | 3.8 | 1 |  |  |
| 2 | 9,307 | 3.8 | 1.00 [0.84; 1.17] | 0.973 |  |
| 3 | 10,027 | 4.8 | 1.26 [1.10; 1.42] | 0.004 | ** |
| 4 | 11,691 | 5.9 | 1.46 [1.31; 1.62] | <0.001 | *** |
| 5 ‘wealthiest’ | 14,107 | 5.6 | 1.42 [1.25; 1.59] | <0.001 | *** |
| **Occupation** |  |  |  |  |  |
| Not working | 21,467 | 4.5 | 1 |  |  |
| Professional/technical/managerial | 1,857 | 5.8 | 1.04 [0.81; 1.27] | 0.733 |  |
| Clerical | 310 | 6.8 | 1.10 [0.62; 1.57] | 0.706 |  |
| Sales | 3,619 | 9.0 | 1.38 [1.23; 1.52] | <0.001 | *** |
| Agricultural – self employed | 7,975 | 4.4 | 0.90 [0.75; 1.05] | 0.174 |  |
| Agricultural – employee | 7,055 | 3.2 | 0.72 [0.55; 0.89] | <0.001 | *** |
| Household/domestic | 1,053 | 6.3 | 1.19 [0.91; 1.46] | 0.225 |  |
| Services | 2,154 | 7.5 | 1.38 [1.31; 1.57] | <0.001 | *** |
| Skilled manual | 3,474 | 6.1 | 1.15 [0.98; 1.33] | 0.114 |  |
| Unskilled manual | 4,008 | 4.6 | 0.91 [0.72; 1.09] | 0.280 |  |
| Don’t know | 262 | 3.1 | 1.07 [0.04; 1.44] | 0.348 |  |
| Sex | | | | | |
| Male | 27,698 | 4.0 | 1 |  |  |
| Female | 25,536 | 6.0 | 2.02 [1.92; 2.12] | <0.001 | *** |
| Age (per 5-year age group) | | | | | |
| 15-19 | 25,586 | 3.0 | 1 |  |  |
| 20-24 | 20,548 | 6.7 | 2.32 [2.22; 2.42] | <0.001 | *** |
| 25-29 | 7,100 | 7.0 | 2.98 [2.83; 4.13] | <0.001 | *** |
|  |  |  |  |  |  |
| *Model summary: AIC = 19,723.6; BIC = 19,991.0; logLik = -9,839.8; DF = 53,212; Deviance = 19,679.6*  *Random effect (CLUST.ID): Variance = 0.763; SD = 0.874* | | | | | |
|  | | | | | |

Significance codes: 0 ‘***’ 0.001 ‘**’ 0.01 ‘*’ 0.05 ‘.’ 0.1 ‘ ’ 1

*N* = Number of observations, aOR = Adjusted Odds Ratio, CI = Confidence Interval, AIC = Akaike Information Criterion, BIC = Bayesian Information Criterion, logLik = log likelihood, DF = Degrees of Freedom, SD = Standard Deviation, N/A = Not Applicable, ‘-’ = Covariate not present in regression model
