# Supplementary material for: Mapping and characterising areas with high levels of HIV transmission in sub-Saharan Africa: A geospatial analysis of national survey data
Source: PLoS Med. 2020 Mar 6;17(3):e1003042. doi: 10.1371/journal.pmed.1003042 (PMC7059914; doi:10.1371/journal.pmed.1003042)
Supplement: S7 Table — Data obtained through (https://dhsprogram.com/). (DOCX) [file pmed.1003042.s023.docx]

**S7 Table. Multiple multilevel logistic regression model of HIV status and environmental variables in young adults (women 15-24 years and men 15-29 years of age) for seven countries of Eastern and Southern Africa, adjusted for age and sex.** Data obtained through (<https://dhsprogram.com/>).

|  | **Young adults** | | | | |
| --- | --- | --- | --- | --- | --- |
| **Covariate** | ***N*** | **HIV prevalence (%)** | **aOR [95% CI]** | **p-value** | |
| **Type of place of residence** |  |  |  |  |  |
| Urban | 18,519 | 7.2 | 1 |  |  |
| Rural | 34,715 | 3.7 | 0.53 [0.36; 0.69] | <0.001 | *** |
| **Population density (km^2^)** |  |  |  |  |  |
| ≤25 | 12,673 | 5.3 | 1.45 [1.26; 1.64] | <0.001 | *** |
| >25 - ≤50 | 6,996 | 4.2 | 1.20 [0.98; 1.41] | 0.099 | . |
| >50 - ≤100 | 4,650 | 3.2 | 0.90 [0.66; 1.15] | 0.414 |  |
| >100 - ≤250 | 8,182 | 4.0 | 1 |  |  |
| >250 - ≤500 | 6,602 | 4.4 | 0.91 [0.69; 1.12] | 0.376 |  |
| >500 | 14,131 | 6.4 | 1.08 [0.87; 1.30] | 0.461 |  |
| **Proximity nearest major city (km)** |  |  |  |  |  |
| ≤10 | 6,114 | 5.9 | 0.77 [0.55; 1.00] | 0.026 | * |
| >10 - ≤50 | 6,221 | 4.5 | 0.91 [0.73; 1.09] | 0.313 |  |
| >50 - ≤100 | 7,986 | 5.1 | 1.16 [1.01; 1.32] | 0.052 | . |
| >100 - ≤500 | 31,879 | 4.7 | 1 |  |  |
| >500 | 1,034 | 9.4 | 1.98 [1.65; 2.30] | <0.001 | *** |
| **Enhanced vegetation index (EVI)** |  |  |  |  |  |
| ≤51 (water bodies, no DHS clusters here) | N/A | N/A | N/A | N/A |  |
| >51 - ≤76 | 495 | 4.1 | 0.52 [-0.10; 1.13] | 0.035 | * |
| >76 - ≤102 | 1,724 | 4.1 | 0.63 [0.31; 0.96] | 0.006 | ** |
| >102 - ≤137 | 9,405 | 5.1 | 0.88 [0.72; 1.04] | 0.106 |  |
| >137 - ≤181 | 22,949 | 5.0 | 1 |  |  |
| >181 - ≤250 | 18,661 | 4.9 | 1.16 [1.03; 1.29] | 0.024 | * |
| **Global human footprint (GHF) (%)** |  |  |  |  |  |
| ≤17 | 2,028 | 4.3 | 0.96 [0.67; 1.25] | 0.800 |  |
| >17 - ≤29 | 15,924 | 3.8 | 1 |  |  |
| >29 - ≤41 | 18,675 | 4.3 | 1.28 [1.14; 1.43] | <0.001 | *** |
| >41 - ≤57 | 6,359 | 6.7 | 1.43 [1.20; 1.66] | 0.003 | ** |
| >57 - ≤100 | 10,248 | 6.9 | 1.68 [1.41; 1.94] | <0.001 | *** |
| Sex | | | | | |
| Male | 27,698 | 4.0 | 1 |  |  |
| Female | 25,536 | 6.0 | 2.08 [1.98; 2.18] | <0.001 | *** |
| Age (per 5-year age group) | | | | | |
| 15-19 | 25,586 | 3.0 | 1 |  |  |
| 20-24 | 20,548 | 6.7 | 2.30 [2.20; 2.39] | <0.001 | *** |
| 25-29 | 7,100 | 7.0 | 3.92 [3.78; 4.06] | <0.001 | *** |
|  |  |  |  |  |  |
| *Model summary: AIC = 19,138.0; BIC = 19,351.2; logLik = -9,545.0; DF = 53,210; Deviance = 19,090.0*  *Random effect (CLUST.ID): Variance = 0.528; SD = 0.727* | | | | | |
|  | | | | | |

Significance codes: 0 ‘***’ 0.001 ‘**’ 0.01 ‘*’ 0.05 ‘.’ 0.1 ‘ ’ 1

*N* = Number of observations, aOR = Adjusted Odds Ratio, CI = Confidence Interval, AIC = Akaike Information Criterion, BIC = Bayesian Information Criterion, logLik = log likelihood, DF = Degrees of Freedom, SD = Standard Deviation, N/A = Not Applicable, ‘-’ = Covariate not present in regression model
