# Supplementary material for: Mapping and characterising areas with high levels of HIV transmission in sub-Saharan Africa: A geospatial analysis of national survey data
Source: PLoS Med. 2020 Mar 6;17(3):e1003042. doi: 10.1371/journal.pmed.1003042 (PMC7059914; doi:10.1371/journal.pmed.1003042)
Supplement: S8 Table — Data obtained through (https://dhsprogram.com/). (DOCX) [file pmed.1003042.s024.docx]

**S8 Table. Combined ‘full’ multiple multilevel logistic regression model of HIV status and behavioural, socioeconomic and environmental variables in young adults (women 15-24 years and men 15-29 years of age) for seven countries of Eastern and Southern Africa, adjusted for age and sex.** Data obtained through (<https://dhsprogram.com/>).

|  | **Young adults** | | | | |
| --- | --- | --- | --- | --- | --- |
| **Covariate** | ***N*** | **HIV prevalence (%)** | **aOR [95% CI]** | **p-value** | |
| **Lifetime number of sex partners** |  |  |  |  |  |
| None | 16,532 | 2.4 | 1 |  |  |
| 1-3 | 27,739 | 5.6 | 1.53 [1.40; 1.66] | <0.001 | *** |
| 4-9 | 7,002 | 7.3 | 2.30 [2.14; 2.46] | <0.001 | *** |
| 10+ | 1,961 | 8.5 | 2.87 [2.65; 3.09] | <0.001 | *** |
| **STI or signs of STI past 12 months** |  |  |  |  |  |
| No | 49,351 | 4.6 | 1 |  |  |
| Yes | 3,883 | 9.1 | 1.57 [1.44; 1.70] | <0.001 | *** |
| **Circumcised (only men)** |  |  |  |  |  |
| No | 17,250 | 4.6 | 1 |  |  |
| Yes | 10,448 | 2.9 | 0.61 [0.47; 0.76] | <0.001 | *** |
| **Education** |  |  |  |  |  |
| No education | 2,410 | 5.2 | 0.99 [0.79; 1.20] | 0.947 |  |
| Primary | 25,013 | 4.8 | 1 |  |  |
| Secondary | 23,956 | 5.1 | 0.84 [0.74; 0.93] | <0.001 | *** |
| Higher | 1,855 | 4.5 | 0.52 [0.27; 0.77] | <0.001 | *** |
| **Type of place of residence** |  |  |  |  |  |
| Urban | 18,519 | 7.2 | 1 |  |  |
| Rural | 34,715 | 3.7 | 0.46 [0.29; 0.62] | <0.001 | *** |
| **Enhanced vegetation index (EVI)** |  |  |  |  |  |
| ≤51 (water bodies, no DHS clusters here) | N/A | N/A | N/A | N/A |  |
| >51 - ≤76 | 495 | 4.1 | 0.52 [-0.12; 1.15] | 0.041 | * |
| >76 - ≤102 | 1,724 | 4.1 | 0.65 [0.32; 0.97] | 0.009 | ** |
| >102 - ≤137 | 9,405 | 5.1 | 0.85 [0.70; 1.01] | 0.042 | * |
| >137 - ≤181 | 22,949 | 5.0 | 1 |  |  |
| >181 - ≤250 | 18,661 | 4.9 | 1.17 [1.04; 1.29] | 0.015 | * |
| **Global human footprint (GHF) (%)** |  |  |  |  |  |
| ≤17 | 2,028 | 4.3 | 1.13 [0.84; 1.41] | 0.418 |  |
| >17 - ≤29 | 15,924 | 3.8 | 1 |  |  |
| >29 - ≤41 | 18,675 | 4.3 | 1.15 [1.01; 1.29] | 0.055 | . |
| >41 - ≤57 | 6,359 | 6.7 | 1.23 [1.02; 1.44] | 0.051 | . |
| >57 - ≤100 | 10,248 | 6.9 | 1.28 [1.06; 1.50] | 0.024 | * |
| Sex | | | | | |
| Male | 27,698 | 4.0 | 1 |  |  |
| Female | 25,536 | 6.0 | 2.04 [1.91; 2.15] | <0.001 | *** |
| Age (per 5-year age group) | | | | | |
| 15-19 | 25,586 | 3.0 | 1 |  |  |
| 20-24 | 20,548 | 6.7 | 1.82 [1.72; 1.93] | <0.001 | *** |
| 25-29 | 7,100 | 7.0 | 2.75 [2.60; 2.90] | <0.001 | *** |
|  |  |  |  |  |  |
| *Model summary: AIC = 19,369.8; BIC = 19,565.2; logLik = -9,662.9; DF = 53,212; Deviance = 19,325.8*  *Random effect (CLUST.ID): Variance = 0.688; SD = 0.830* | | | | | |
|  | | | | | |

Significance codes: 0 ‘***’ 0.001 ‘**’ 0.01 ‘*’ 0.05 ‘.’ 0.1 ‘ ’ 1

*N* = Number of observations, aOR = Adjusted Odds Ratio, CI = Confidence Interval, AIC = Akaike Information Criterion, BIC = Bayesian Information Criterion, logLik = log likelihood, DF = Degrees of Freedom, SD = Standard Deviation, N/A = Not Applicable, ‘-’ = Covariate not present in regression model
