# Supplementary material for: Mapping and characterising areas with high levels of HIV transmission in sub-Saharan Africa: A geospatial analysis of national survey data
Source: PLoS Med. 2020 Mar 6;17(3):e1003042. doi: 10.1371/journal.pmed.1003042 (PMC7059914; doi:10.1371/journal.pmed.1003042)
Supplement: S9 Table — Data obtained through (https://dhsprogram.com/). (DOCX) [file pmed.1003042.s025.docx]

**S9 Table. Combined ‘full’ multiple multilevel model as modified Poisson regression (with robust variance) of HIV status and behavioural, socioeconomic and environmental variables in young adults (women 15-24 years and men 15-29 years of age) for seven countries of Eastern and Southern Africa, adjusted for age and sex.** Data obtained through (<https://dhsprogram.com/>).

|  | **Young adults** | | | | |
| --- | --- | --- | --- | --- | --- |
| **Covariate** | ***N*** | **HIV prevalence (%)** | **aRR [95% CI]** | **p-value** | |
| **Lifetime number of sex partners** |  |  |  |  |  |
| None | 16,532 | 2.4 | 1 |  |  |
| 1-3 | 27,739 | 5.6 | 1.52 [1.39; 1.64] | <0.001 | *** |
| 4-9 | 7,002 | 7.3 | 2.18 [2.03; 2.34] | <0.001 | *** |
| 10+ | 1,961 | 8.5 | 2.63 [2.42; 2.84] | <0.001 | *** |
| **STI or signs of STI past 12 months** |  |  |  |  |  |
| No | 49,351 | 4.6 | 1 |  |  |
| Yes | 3,883 | 9.1 | 1.46 [1.34; 1.58] | <0.001 | *** |
| **Circumcised (only men)** |  |  |  |  |  |
| No | 17,250 | 4.6 | 1 |  |  |
| Yes | 10,448 | 2.9 | 0.64 [0.50; 0.77] | <0.001 | *** |
| **Education** |  |  |  |  |  |
| No education | 2,410 | 5.2 | 0.98 [0.79; 1.17] | 0.812 |  |
| Primary | 25,013 | 4.8 | 1 |  |  |
| Secondary | 23,956 | 5.1 | 0.85 [0.77; 0.94] | <0.001 | *** |
| Higher | 1,855 | 4.5 | 0.54 [0.30; 0.78] | <0.001 | *** |
| **Type of place of residence** |  |  |  |  |  |
| Urban | 18,519 | 7.2 | 1 |  |  |
| Rural | 34,715 | 3.7 | 0.50 [0.35; 0.65] | <0.001 | *** |
| **Enhanced vegetation index (EVI)** |  |  |  |  |  |
| ≤51 (water bodies, no DHS clusters here) | N/A | N/A | N/A | N/A |  |
| >51 - ≤76 | 495 | 4.1 | 0.61 [0.05; 1.17] | 0.087 | . |
| >76 - ≤102 | 1,724 | 4.1 | 0.70 [0.41; 1.00] | 0.019 | * |
| >102 - ≤137 | 9,405 | 5.1 | 0.87 [0.73; 1.01] | 0.055 | . |
| >137 - ≤181 | 22,949 | 5.0 | 1 |  |  |
| >181 - ≤250 | 18,661 | 4.9 | 1.17 [1.05; 1.22] | 0.007 | ** |
| **Global human footprint (GHF) (%)** |  |  |  |  |  |
| ≤17 | 2,028 | 4.3 | 1.09 [0.82; 1.35] | 0.537 |  |
| >17 - ≤29 | 15,924 | 3.8 | 1 |  |  |
| >29 - ≤41 | 18,675 | 4.3 | 1.13 [1.00; 1.26] | 0.062 | . |
| >41 - ≤57 | 6,359 | 6.7 | 1.21 [1.02; 1.39] | 0.053 | . |
| >57 - ≤100 | 10,248 | 6.9 | 1.25 [1.05; 1.44] | 0.023 | * |
| Sex | | | | | |
| Male | 27,698 | 4.0 | 1 |  |  |
| Female | 25,536 | 6.0 | 2.03 [1.94; 2.12] | <0.001 | *** |
| Age (per 5-year age group) | | | | | |
| 15-19 | 25,586 | 3.0 | 1 |  |  |
| 20-24 | 20,548 | 6.7 | 1.72 [1.62; 1.82] | <0.001 | *** |
| 25-29 | 7,100 | 7.0 | 2.51 [2.36; 2.65] | <0.001 | *** |
|  |  |  |  |  |  |
| *Model summary: AIC = 19,685.2; BIC = 19,880.7; logLik = -9,820.6; DF = 53,212; Deviance = 19,641.2*  *Random effect (CLUST.ID): Variance = 0.473; SD = 0.688* | | | | | |
|  | | | | | |

Significance codes: 0 ‘***’ 0.001 ‘**’ 0.01 ‘*’ 0.05 ‘.’ 0.1 ‘ ’ 1

*N* = Number of observations, aRR = Adjusted Relative Risk, CI = Confidence Interval, AIC = Akaike Information Criterion, BIC = Bayesian Information Criterion, logLik = log likelihood, DF = Degrees of Freedom, SD = Standard Deviation, N/A = Not Applicable, ‘-’ = Covariate not present in regression model
