# Supplementary material for: Mapping and characterising areas with high levels of HIV transmission in sub-Saharan Africa: A geospatial analysis of national survey data
Source: PLoS Med. 2020 Mar 6;17(3):e1003042. doi: 10.1371/journal.pmed.1003042 (PMC7059914; doi:10.1371/journal.pmed.1003042)
Supplement: S10 Table — Data obtained through (https://dhsprogram.com/). (DOCX) [file pmed.1003042.s026.docx]

**S10 Table. Combined ‘full’ multiple multilevel logistic regression model of HIV status and behavioural, socioeconomic and environmental variables in young adults (women 15-24 years and men 15-29 years of age) for seven countries of Eastern and Southern Africa, adjusted for age, sex, and country.** Data obtained through (<https://dhsprogram.com/>).

|  | **Young adults** | | | | |
| --- | --- | --- | --- | --- | --- |
| **Covariate** | ***N*** | **HIV prevalence (%)** | **aOR [95% CI]** | **p-value** | |
| **Lifetime number of sex partners** |  |  |  |  |  |
| None | 16,532 | 2.4 | 1 |  |  |
| 1-3 | 27,739 | 5.6 | 1.47 [1.35; 1.60] | <0.001 | *** |
| 4-9 | 7,002 | 7.3 | 2.18 [2.02; 2.35] | <0.001 | *** |
| 10+ | 1,961 | 8.5 | 2.66 [2.44; 2.88] | <0.001 | *** |
| **STI or signs of STI past 12 months** |  |  |  |  |  |
| No | 49,351 | 4.6 | 1 |  |  |
| Yes | 3,883 | 9.1 | 1.69 [1.56; 1.86] | <0.001 | *** |
| **Circumcised (only men)** |  |  |  |  |  |
| No | 17,250 | 4.6 | 1 |  |  |
| Yes | 10,448 | 2.9 | 0.68 [0.53; 0.83] | <0.001 | *** |
| **Education** |  |  |  |  |  |
| No education | 2,410 | 5.2 | 1.01 [0.81; 1.21] | 0.923 |  |
| Primary | 25,013 | 4.8 | 1 |  |  |
| Secondary | 23,956 | 5.1 | 0.77 [0.67; 0.86] | <0.001 | *** |
| Higher | 1,855 | 4.5 | 0.49 [0.24; 0.74] | <0.001 | *** |
| **Type of place of residence** |  |  |  |  |  |
| Urban | 18,519 | 7.2 | 1 |  |  |
| Rural | 34,715 | 3.7 | 0.57 [0.40; 0.73] | <0.001 | *** |
| **Global human footprint (GHF) (%)** |  |  |  |  |  |
| ≤17 | 2,028 | 4.3 | 0.95 [0.67; 1.23] | 0.743 |  |
| >17 - ≤29 | 15,924 | 3.8 | 1 |  |  |
| >29 - ≤41 | 18,675 | 4.3 | 1.25 [1.11; 1.39] | 0.001 | ** |
| >41 - ≤57 | 6,359 | 6.7 | 1.25 [1.05; 1.45] | 0.029 | * |
| >57 - ≤100 | 10,248 | 6.9 | 1.16 [0.95; 1.36] | 0.162 |  |
| Sex | | | | | |
| Male | 27,698 | 4.0 | 1 |  |  |
| Female | 25,536 | 6.0 | 2.05 [1.92; 2.15] | <0.001 | *** |
| Age (per 5-year age group) | | | | | |
| 15-19 | 25,586 | 3.0 | 1 |  |  |
| 20-24 | 20,548 | 6.7 | 1.85 [1.74; 1.95] | <0.001 | *** |
| 25-29 | 7,100 | 7.0 | 2.81 [2.66; 2.96] | <0.001 | *** |
| Country | | | | | |
| Kenya | 3,286 | 3.8 | 1 |  |  |
| Malawi | 7,097 | 3.6 | 0.88 [0.63; 1.13] | 0.304 |  |
| Mozambique | 4,129 | 8.6 | 1.84 [1.58; 2.09] | <0.001 | *** |
| Tanzania | 8,262 | 1.8 | 0.49 [0.22; 0.76] | <0.001 | *** |
| Uganda | 9,297 | 3.6 | 0.82 [0.57; 1.07] | 0.115 |  |
| Zambia | 13,416 | 7.3 | 1.87 [1.64; 2.10] | <0.001 | *** |
| Zimbabwe | 7,747 | 5.6 | 1.55 [1.31; 1.80] | <0.001 | *** |
|  |  |  |  |  |  |
| *Model summary: AIC = 19,138.0; BIC = 19,351.2; logLik = -9,545.0; DF = 53,210; Deviance = 19,090.0*  *Random effect (CLUST.ID): Variance = 0.5277; SD = 0.7265*  *Marginal R^2^ = 17.9, conditional R^2^ = 29.3* | | | | | |
|  | | | | | |

Significance codes: 0 ‘***’ 0.001 ‘**’ 0.01 ‘*’ 0.05 ‘.’ 0.1 ‘ ’ 1

*N* = Number of observations, aOR = Adjusted Odds Ratio, CI = Confidence Interval, AIC = Akaike Information Criterion, BIC = Bayesian Information Criterion, logLik = log likelihood, DF = Degrees of Freedom, SD = Standard Deviation, N/A = Not Applicable, ‘-’ = Covariate not present in regression model
