# Supplementary material for: Mapping and characterising areas with high levels of HIV transmission in sub-Saharan Africa: A geospatial analysis of national survey data
Source: PLoS Med. 2020 Mar 6;17(3):e1003042. doi: 10.1371/journal.pmed.1003042 (PMC7059914; doi:10.1371/journal.pmed.1003042)
Supplement: S11 Table — (DOCX) [file pmed.1003042.s027.docx]

**S11 Table. Overview of the heterogeneity (R^2^) explained by the full final logistic regression model (see S10 Table), for each country separately.**

| **Country** | **Conditional R^2^ – total heterogeneity explained by model (%)** | **Marginal R^2^ – heterogeneity explained by included fixed effects (%)** | |
| --- | --- | --- | --- |
| Kenya | 45.0 | | 35.1 |
| Malawi | 31.3 | | 16.2 |
| Mozambique | 27.3 | | 17.8 |
| Tanzania | 36.0 | | 20.8 |
| Uganda | 23.5 | | 15.7 |
| Zambia | 31.6 | | 21.5 |
| Zimbabwe | 17.6 | | 13.0 |
