# Supplementary material for: Mapping and characterising areas with high levels of HIV transmission in sub-Saharan Africa: A geospatial analysis of national survey data
Source: PLoS Med. 2020 Mar 6;17(3):e1003042. doi: 10.1371/journal.pmed.1003042 (PMC7059914; doi:10.1371/journal.pmed.1003042)
Supplement: Attachment — Submitted filename: FINAL_PLOS Medicine_reviewers_comments.docx [file pmed.1003042.s028.docx]

**Requests from the editors:**

1. *Our academic editor commented that the term "hotspot" is not favoured by people with HIV, and we ask you to amend this.*

Thank you for raising this, we have now changed all mentions of ‘hotspots’ to ‘high prevalence areas’, or ‘areas with high levels of HIV transmission’.

2. *Also, it was suggested that "the framing could be that by addressing areas of high incidence we are addressing inequities rather than focusing on areas perceived to be driving the epidemic".*

An interesting point, and we understand the sensitivities here. Our analysis cannot specifically demonstrate that these areas are indeed driving the epidemic. Identification of high transmission areas is only an instrument to better understand the (social) processes underlying the spread of HIV.

We have thus revised several sections in our manuscript to ensure that we talk about areas in the context of specific inequalities that give rise to high HIV risk (e.g. high-risk behaviour, poor access to services), thereby justifying targeted interventions, rather than the areas being drivers of the epidemic.

3. *In your abstract, please specify the range of years that the source datasets refer to. Also, please add summary demographic details for study participants.*

We have added the requested information as follows: abstract, methods and findings subsection:

“We used geolocated survey data from the most recent USAID Demographic and Health Surveys and AIDS Indicator Surveys (collected between 2008-2009 and 2015-2016), which included about 113 thousand adults, of which about 53 thousand young adults (27 thousand women, 28 thousand men), from over 3,500 sample locations.”

We also added detailed summary demographics to the main text (results, 1^st^ paragraph):

“Our study included 112,785 adults, of which 53,234 young adults (25,536 women, 27,698 men), from 3,665 different sample locations throughout the seven countries.”

4. *Please add a new final sentence to the "methods and findings" subsection of your abstract to summarize the study's main limitations.*

As requested, we have added the following to the methods and findings subsection of the abstract: “Main limitations were the inability to infer causality due to the cross-sectional nature of the surveys, and the likely under-sampling of key populations in the surveys.”

5. *At line 40, please start the "Conclusions" subsection with "In this study, we found that ..." or similar.*

Done.

6. After the abstract, we ask you to add a new and accessible "author summary" section in non-identical prose. You may find it helpful to consult one or two recent research papers published in PLOS Medicine to get a sense of the preferred style.

Done

7. *In your methods section, please briefly mention the situation with ethics approval (e.g., not required for this analysis).*

Many thanks. Ethical approval was indeed not required for this study, as we performed analyses on anonymised, open access DHS data. We added the following statement:

Methods, last paragraph:

“All the utilised DHS and AIS datasets are publicly available, and the DHS programme de‐identifies all data before making them available to the public. The geospatial data (WorldPop, NASA) do not contain variables at the level of human subjects. Therefore, this work did not require ethical approval.”

8. *Early in the methods section of your main text, please state whether the study had a protocol or prespecified analysis plan, and if so, attach the relevant document(s) as a supplementary file (referred to in the methods). Please highlight analyses that were not prespecified.*

Our study was essentially exploratory, and thus did not have a formal prespecified analysis plan.

We added the following sentence at the start of the section “statistical analysis” (methods, 5^th^ paragraph): “Our study was explorative in nature, and we did not have a formal prespecified analysis plan.”

9. *Please add a completed checklist for the most appropriate reporting guideline, which may be RECORD, as a supplementary file (again referred to in your methods section. In the checklist, individual items should be referred to by section (e.g., "Methods") and paragraph number rather than by line or page numbers, as the latter generally change in the event of publication.*

We added the completed RECORD checklist to the supplements (S2 Table) and referred to the checklist in the methods section of the manuscript, 7^th^ paragraph: “Reporting of study design and analysis followed STROBE RECORD guidelines (S2 Table) [1].”.

10. *When discussing your conclusions, e.g. in the first paragraph of your Discussion section, please ensure that findings are referred to consistently in the past tense (e.g., "alternated" at line 268) with conclusions in the present tense (e.g., "Our findings show ..." at line 266).*

Thank you for pointing out these typos, we have carefully re-read our manuscript and corrected the verb tense mistakes where needed.

11. *Please avoid claims of primacy (e.g., at line 317), and where used add "to our knowledge" or similar.*

Done

12. *Please remove "(80-)" from reference 17.*

Done

**Comments from the reviewers:**

***** Reviewer #1:**

*I confine my remarks to statistical aspects of this paper. I have a couple of fairly major issues to resolve before I can recommend publication, but I think there's a good paper here.*

We thank the reviewer for his positive assessment of our manuscript, and appreciate his comprehensive assessment of our statistical analyses.

1. *My first question is what was done to avoid finding patterns in pure noise? How can we be sure that these patterns are not just things found by the power of the method?*

We agree with the reviewer that, in principle, finding random noise is always a risk in explorative statistical analyses. However, we feel that there are several reasons why the risk of mistaking random noise for a signal is extremely low in our study. Specifically, also because we had similar concerns, we selected only a small number of candidate variables (13 out of the total of more than 250 from a full DHS survey), all based on substantive knowledge, i.e. variables that are specifically of interest and likely associated with HIV, based on findings from previous studies. The very large sample size (*n* = 53,234) and the directions and magnitudes of association between the included covariates and HIV found in our study, which are all in line with findings from previous studies [2–5], make finding patterns of pure noise unlikely.

In response to the concerns of the reviewer, we have now also performed a cross-validation on non-random subsamples of the data to assess the internal validity of our estimates. We split the data by country and tested how much heterogeneity (indicated by the conditional R^2^) was explained by the final regression model, as suggested by Steyerberg and colleagues [6,7]. Reassuringly we found that, despite the differences in underlying epidemic and scale-up of interventions across countries, the conditional R^2^ of the model was strikingly similar for six out of the eight countries in our analysis, ranging between 24% and 36% (compared to 30% in the combined analysis). Only the conditional R^2^ for Kenya (45%) and Zimbabwe (17%) deviated a little more from the combined model, yet not to an alarming extend.

We have added the following sentences to the methods section (last paragraph):

“Next to the main analyses, we also performed an internal validation of our regression models, and an external validation of our kriging results. The internal validation was done using non-random cross-validation [6,7] to check for each country individually how much heterogeneity (indicated by the conditional R^2^) was explained by the final regression model.”.

We have added the following sentences to the discussion (6^th^ paragraph):

“Fourth, explorative statistical analyses always run the risk of identifying patterns of random noise [8]. However, we believe this risk to be extremely low in our study, due to the very large sample size (*n* = 53,234), the rigid pre-selection of variables based on substantive knowledge, and the directions and magnitudes of association between the included covariates and HIV found in our study are in line with of findings from previous studies [2–5]. In addition, we performed non-random cross-validation by testing the final fitted model for each country separately. Reassuringly we found that, despite the differences in underlying epidemic and scale-up of interventions across countries, the conditional R^2^ of the model was strikingly similar for six out of the eight countries in our analysis, ranging between 23.5% and 36.0% (compared to 29.6% in the main analysis). Only the conditional R^2^ for Kenya (45.0%) and Zimbabwe (17.6%) deviated a little bit more from the combined model, yet not to an alarming extend (S12 Table).”

2. *The second big thing is the model formation. The authors did bivariate screening followed by stepwise methods. Both of these are problematic. All of the output from such methods is wrong: Parameter estimates are biased away from 0, p-values are too small, standard errors are too small (see Harrell **Regression Modelling Strategies* for lots of details).  It would be better to use substantive knowledge to build a model. If the authors insist on not using their knowledge and want an automatic method, the LASSO is probably the best of the readily available methods.*

We thank the reviewer for this insightful point, which is essentially the same as previous point. It is important to realize that our analyses are explorative in nature, and did not aim to develop comprehensive predictive statistical models of HIV risk. Precisely for this reason, we did use substantive knowledge to select the covariates tested in our regression models: we selected only 13 out of the more than 250 variables in DHS surveys because of their known association with HIV, and we always incorporated age and sex, regardless of whether it was significantly associated with HIV. We assumed that all variables played some, perhaps very minor, roles, i.e. no null hypothesis was really true. What we tried however was to identify variables that did go some way in understanding observed heterogeneities in this unique pandemic/epidemic, which is not what the statistical methods used in our study were developed for, but nevertheless proved useful for this task.

The LASSO and other regularization methods were developed for improving predictive accuracy which is not our aim.

To improve the clarity of our approach, we added the following to the methods section (3^rd^ paragraph): “A typical DHS survey dataset contains over 250 variables. For the purpose or our study, we only extracted individual-level candidate variables that were deemed of interest, based on findings from previous studies [2,4,5,9–12]”

*More minor points*

1. *The authors should list all the variables and how they were measured. Not just the ones that wound up in the model, but all the ones that were available*

An excellent suggestion. We added a supplementary table that consistently describes all the variables used in our analysis including the sources, how they are measured, and what they mean (see S1 Table). We refer to this table at the end of the 3^rd^ paragraph of the methods section: “An overview and description of all included variables can be found in the supplementary information (S1 Table).”.

2. *Line 139   "Multivariate" should be "multiple"*

Thank you for noting this, we have changed “multivariate” to “multiple” throughout the manuscript.

3. *Line 144 Age and sex should be fixed effects*

Indeed, corrected.

4. *Line 153 and another places R^2 is mistyped as R2*

We double checked our manuscript, and only found one instance where this was mistyped, in reference 11. To our knowledge, all other instances were noted as R^2^ (i.e. R^2) and not R2. We apologize if manuscript conversions during the submission process may have removed the superscript notation of R^2^.

5. *Line 158 I don't understand how random effects were translated into MOR. First, random effects are usually nuisance variables that you are not particularly interested in. Second, the standard errors for random effects are very poorly estimated (so much that some programs don't even print them) so I think the authors mean fixed effects. Third, I don't know how the translation was done.*

We fully agree with the reviewer that random effects are, in general, nuisance variables that are rarely of interest, and we can certainly understand the confusion. However, in our analysis we used geographical random effects as a way of identifying geographical variation that is not “explained” by observed geographical or individual characteristics. By comparing heterogeneity in random effects estimates, we can essentially compare unexplained geographical variation at the cluster level before and after incorporating certain fixed effects with known associations with HIV.

We have now explained this more extensively in our revised manuscript, including an explanation how MORs were calculated (‘methods’ section, ‘statistical analyses’ subsection, 2^nd^ paragraph): “We translated variance of random effects into Median Odds Ratios (MORs) as an indicator geographical heterogeneity. For each model, the MOR would be equal to 1.0 if there were no differences in probability to being HIV infected per sample location, and can be interpreted as the increase in (median) HIV risk that is associated with moving from a location with a low random effect to a location with a high random effect.”

Further information can also be found in the article by Merlo et al. (2006) [13], referenced in the manuscript directly after the previous part (‘methods’ section, ‘statistical analyses’ subsection, 2^nd^ paragraph): “See elsewhere for a more detailed explanation [13].”

The text in the results section regarding the MORs has been adapted to: “The MOR of the nested model is 2.41, whereas the MOR of the final model is 1.94. This illustrates that, although some of the location-level heterogeneity is captured by the model fixed effect covariates, almost two-thirds of the heterogeneity in HIV prevalence at sample locations could still not be explained by the covariates in the model. Overall, environmental fixed effects reduce the location-level heterogeneity more than sexual behavioural and socioeconomic fixed effects (MORs of 1.95, 2.35, and 2.30 respectively).”.

6. *Figure 1 How were the outliers dealt with?*

Good question. We did not treat outliers any differently in our analyses. Outliers of HIV prevalence at sample locations (as displayed in Figure 1 of the manuscript) could likely exist due to two reasons. On the one hand, they can result from underlying area characteristics driving the local HIV epidemic, such as an active sex industry. On the other hand, outliers could be inaccurate estimates due to small sample sizes at the sample location, e.g. increasing the weight of HIV positive individuals within the sample. We checked the sample sizes of outliers of high HIV prevalence levels and no outliers showed extremely low sample sizes, making the first explanation of their existence more plausible. Since we are primarily interested in these areas, we kept these crude estimates in our analyses.

*Peter Flom*

***** Reviewer #2:**

*This work attempts to map 'hotspots' for young adults (proxy for higher transmission hotspots for targeting) in seven countries in Eastern and Southern Africa, specifically identify significant clustering and associated determinants potentially explaining the underlying heterogeneity observed. The work I believe makes a sufficient contribution/step forward to the existing body of literature in this area, is well written and should be useful for policy makers in these countries. I have some comments and suggestions below that need to be addressed before the paper can be accepted.*

We thank the reviewer for her/his positive assessment of our manuscript.

*General:  Identifying the hotspots is the first natural step as currently presented in the paper. However, the current detailed description of explained versus unexplained heterogeneity explained for the larger covariate groupings in the main results text is not in a policy maker friendly format and needs to be revised to maximise the readability/usability of these results. It would be more useful to present key findings highlighting which risk factors or covariates are the most attributable both within and across countries to assist with geographic/locally tailored intervention packages.*

We much appreciate the reviewers’ comment, and agree that the ultimate goal of our work should be to achieve better control of the epidemic through improved policy. The main objective of our paper was to show that we can identify general patterns of clustering of HIV transmission using HIV prevalence data in young adults, and whether there are common individual level or environmental denominators that can explain this heterogeneity. Although we did find some important general risk factors suggesting that especially areas with high levels of economic activity and sex work are at increased risk, the relative importance of each risk factor is likely context specific, making it essential for policy makers to directly evaluate the behavioural and socio-economic context, and the interventions already in place at a specific high-risk setting, to tailor interventions appropriately. We would like to therefore caution to directly translate our regression coefficients into policy.

We added the following to the manuscript (discussion, 4th paragraph):

“Ultimately, our results demonstrate that the decade-old mantra of “know your epidemic, know your response” [14] is still highly relevant for SSA. Our study found important common denominators that are associated with increased HIV risk in areas with high levels of transmission, but it will be essential for policy makers to specifically evaluate the behavioural and socio-economic context, and the interventions already in place at a specific high-risk setting to tailor interventions appropriately.”

Nevertheless, we also agree with the reviewer that providing more detailed results of key covariates would be helpful, and we further specified the reported associations between HIV and the included covariates that we included in our regression models. In the results section of the manuscript (3^rd^ paragraph), we adapted the regression results as follows:

“According to the resulting best fitting multiple multilevel logistic regression model on the association between HIV status and sexual behavioural variables in young adults, the following variables were strongly associated with being infected with HIV: 10 or more reported lifetime sex partners, an STI or STI symptoms over the past 12 months, condom use during last intercourse, and not being circumcised (men only). Educational level, wealth index and occupation were variables associated with HIV in the final socioeconomic model. The highest level of education was most protective (aOR 0.52 [0.26; 0.78], p-value <0.001), while being from asset quintile 4, the second wealthiest quintile, was associated with the highest risk of HIV (aOR 1.46 [1.31; 1.62], p-value <0.001). Several variables were significantly associated with HIV in the environmental model. Young adults living in rural sample locations were less likely to be infected with HIV than those with urban residence: in cities the overall HIV prevalence among adults was over 7%, compared to below 4% in rural settings. Also, population density, proximity to nearest major city, EVI and GHF at location of a DHS cluster showed a significant association with HIV. HIV prevalence levels were highest (about 6%) in areas with the highest population density (more than 500 people per km^2^), but was also relatively high (about 5%) in areas with the lowest population density (less than 25 people per km^2^). HIV prevalence levels did not differ considerably between sample locations with different levels of greenness (indicated by the EVI). Living in an area with a relatively high GHF, as proxy for economic activity, was associated with a high HIV risk (aOR 1.68 [1.41; 1.94], p-value <0.001): HIV prevalence levels for the highest two levels of GHF were almost 7%, compared to around 4% at the lower levels. The best fitting combined ‘full’ model contains both behavioural, socioeconomic, and environmental variables: lifetime number of sex partners, STIs, male circumcision, education, type of residence, EVI, and GHF. A complete overview of the bivariate models, nested model (only adjusted for age and sex), best fitting models and final model can be found in the supplementary information (S4 to S9 Tables and S7 Figure). Finally, the combined ‘full’ model was also fitted as a modified Poisson regression model, resulting in aRRs that were comparable to the aORs from the logistic regression model (S10 Table).”

*General: It is also important to present the spatial hotspot/interpolation surface by gender in addition to overall (e.g. SDG 5: Gender equality) to confirm if the same hotspots are identified for both males and females. It may also be worth considering shared component analysis of the gender specific prevalence surfaces to see to which degree they correlate at finer geographic scale and how this varies within and across the countries.*

We agree that focussing on the HIV heterogeneity by sex is important, especially with regard to the current HIV/AIDS targets embedded in the SDGs, primarily to be able to design tailored intervention strategies by sex and enable equal access to healthcare. We want to thank the reviewer for the excellent suggestion on how to present these results. The interpolated HIV prevalence estimates by sex are presented in supplements S6 Figure, where it is shown that heterogeneity exists in both women and men, and that high prevalence areas largely overlap. We now more specifically looked at differences in HIV prevalence between women and men. We illustrated the absolute difference in HIV prevalence by mapping the square root of the squared difference in HIV prevalence (per 5 km^2^ grid cell), and plotted the predicted HIV prevalence (per 5 km grid cell) of women against the predicted HIV prevalence of men.

We included this in the methods section (end of the first paragraph of the ‘statistical analyses’ subsection) and further explained its importance: “We compared the sex-specific surfaces of HIV prevalence by means of mapping the square root of the squared difference in HIV prevalence, to illustrate the absolute differences in HIV prevalence (per 5 km^2^ grid cell) (S6A Figure), and by plotting the predicted HIV prevalence (per 5 km grid cell) of women against the predicted HIV prevalence of men (S6C Figure). Both comparisons show that there are only minor differences in terms of the locations of high HIV prevalence for both sexes.”

*General: Apologies if missed it but there is little or no contextualisation of the results with regards to SDG targets for HIV and how these results are relevant to assessing progress towards these targets and assisting with retailoring of intervention packages across the individual countries in this analysis*.

We appreciate the reviewers’ suggestion and highlighted the important of our study in the light of the SDGs We added the following:

Introduction, 1st paragraph: “Sustainable Development Goal (SDG) 3 “to ensure healthy lives and promote well-being for all at all ages” [15], together with the Joint United Nations Programme on HIV/AIDS (UNAIDS) fast-track strategy, explicitly call to end the pandemic by 2030 [16].”

Discussion, last paragraph (conclusion subsection): “In further reducing HIV transmission in Eastern and Southern Africa, areas of high HIV prevalence in young adults could be priority areas for tailored HIV prevention interventions in line with SDG3 and UNAIDS targets to end the HIV pandemic by 2030.”

*Major comments:*

1. *The analysis utilised data from DHS/AIS surveys, however there is also a wealth of small geographic scale data in the demographic surveillance sites (DSS) in the region and ongoing HIV work in these sites e.g. ALPHA network. I was wondering if the authors considered using prevalence estimates from these DSS as an external validation source for the applied kriging exercise? i.e. does the model predict well at unsampled locations that potentially overlap with DSS.*

This is an interesting suggestion. Even though the generalizability of these population-based cohorts can be debated because of the intensive ongoing research activities at these sites, baseline HIV prevalence levels could give an indication as to whether our approach and observations at those sites are similar.

As per reviewers’ suggestion, we looked for population-based cohorts in the geographical area covered by our study, to compare HIV prevalence for young adults at these sites with our interpolated HIV prevalence estimates. We were not able to extract many HIV prevalence estimates specific for young adults from the ALPHA and other data from small-scale surveillance sites available. We did find a couple of studies that were useful for external validation purposes. A study reporting age-stratified HIV prevalences for the ALPHA network sites for 2011 reports the following prevalences for young adults: Karonga (Malawi) ~3%, Kisesa (Tanzania) ~2%, Kisumu (Kenya) ~11%, Manicaland (Zimbabwe) 8%, Masaka (Uganda) 2%, Rakai (Uganda) ~10% [17]. Only the estimates for the Rakai and Manicaland sites were higher than predicted in our study.

We added the following to the manuscript:

Methods, last paragraph:

“Next to the main analyses, we also performed an internal validation of our regression models, and an external validation of our kriging results. […] For the external validation, we searched peer-reviewed literature reporting on age-stratified HIV prevalence estimates in population-based cohorts, such as those part of the ALPHA network [17], situated in one of the countries of our study. We compared our predicted HIV prevalence among young adults, obtained through kriging, with the estimates from these cohorts.”

Discussion, 2^nd^ paragraph:

“The geospatial patterns of HIV prevalence heterogeneity among adults shown in our study (Figure 2A) are very comparable to patterns shown in other recent studies [9,18], confirming that the utilised methods are suitable for creating reliable estimates. As an external validation for our geospatial patterns of HIV prevalence in young adults, we compared our estimates to the estimates from multiple small-scale surveillance sites from the ALPHA network [17]. We found that most estimates are comparable, but our estimates were lower for the Rakai (Uganda) and Manicaland (Zimbabwe) areas (S8 Figure). Both areas are characterised by well-known high levels of HIV transmission and relatively low numbers of sample locations in the utilised data with varying prevalence levels, leading to underestimation of the HIV prevalence. This indicates that our approach of kriging, in which prevalence at a specific location is estimated by the prevalence in surrounding clusters as a function of the distance between the location and surrounding clusters, may have resulted in an underestimation (i.e. smoothing) of HIV prevalence estimates, especially in high prevalence areas.

2. *Did the authors consider a Bayesian spatial approach/implementation in R-INLA as opposed to ordinary kriging? A spatial Bayesian multivariable formulation including the covariates might then be able to produce uncertainty around predictions at unsampled locations and more efficiently adjust for spatial correlation in nearby sampled clusters as per DHS design. I suspect that a Bayesian approach in INLA (which would be computationally tractable) would perform better than the approaches currently employed in the paper.*

This is an interesting suggestion and we did consider to use a Bayesian spatial approach. Still, in the current analysis we primarily aim to explore which factors explain the HIV heterogeneity rather than directly predict HIV prevalence, and we therefore chose to use relatively simplistic models that we expected to be easy for a broader public to understand, so as to stay “close to the data”. We are aware that kriging is not the only method of spatial interpolation, and that including covariates can improve HIV prevalence predictions. We compared the continuous surface maps from our study to those from existing studies [9,19]. The overall patterns of HIV prevalence heterogeneity and locations of high prevalence areas are similar to the ones in these studies, suggesting that kriging interpolation works well for visualising HIV prevalence in our study. We wanted to show which covariates play a role in explaining the heterogeneity in HIV prevalence among young adults and show their relative importance, and believe that the currently utilised models were most suitable for this purpose.

We added to the discussion, 2^nd^ paragraph: “The geospatial patterns of HIV prevalence heterogeneity among adults shown in our study (Figure 2A) are very comparable to patterns shown in other recent studies in which other methods for spatial interpolation were used [9,18], confirming that our approach was suitable for creating reliable estimates.”

3. *For the risk factor analysis/modelling I can understand not using the original sampling weights from the DHS/AIS. However, when estimating prevalence and generating a smoothed prevalence surface I would suspect that these weights be more important and allow uncertainty intervals (e.g. Figure 1 etc). Did the authors perform a sensitivity analysis to confirm that the surface without weights would be fairly similar to a surface utilising the survey weights? The weights would also be useful if you were to add additional visualisations projecting absolute counts of HIV positive young adults by high versus lower risk clusters. Prevalence without weighted correction may mask underlying population size differences within and across countries.*

The sample weights for DHS/AIS data are officially available for calculating nationally representative estimates for HIV and other variables, as the surveys oversample certain areas to ensure that areas with low population densities are also properly represented. This aspect is of less relevance when the clusters (primary sampling units) are used with their geographical location. In line with these considerations, we chose to use the HIV sample weights to calculate the national HIV prevalence estimates per country, as shown in Figure 1, but not for sample location prevalence levels. Thus, we feel that we have used sample weights where appropriate and ignored them, i.e. in the kriging, where not. Our kriging approach interpolates HIV prevalence in areas based on estimates from the DHS clusters, and thus directly uses locally (i.e. cluster-level) measured HIV prevalence, its uncertainty based on distance and variance in HIV prevalence levels of nearby points. We therefore think that it is more appropriate to use the raw (unweighted) cluster level prevalence estimates, as these purely represent what is actually measured, including sample sizes. The correct way to translate our HIV prevalence estimates into estimates of numbers of people living with HIV in certain areas would be to use location specific population density data, such as those available from WorldPop.org.

We have revised our manuscript to better explain these issues (methods, 1^st^ paragraph):

“Sample weights are incorporated in the DHS to translate unbalanced sampling into national representative data. In our study, we used these sample weights to estimate national HIV prevalence among adults and young adults for all seven countries. For the other analyses, we combined data of multiple countries and explored local level variances, and thus did not use the sampling weights. More details on survey protocols and questionnaires can be found on the DHS website (https://dhsprogram.com/)."

4. *The environmental covariates if I understand it are homogeneous for all individuals within a DHS survey "cluster" or community while those measured at individual level varying at that level. I wonder if this may impact the variable selection approach, especially if income or SES at household level is linked up with GHF (economic activity component) at cluster level.*

This is indeed the case, and an excellent point. This is precisely the reason why we chose to also develop indicator-specific models (i.e. behavioural, socio-economic, and environmental), next to a full regression model. Through these indicator specific models, we could isolate the importance of the various cluster- or individuals level variables. In the combined model, for example, variables that are associated with HIV in the separate models, but also associated with other variables in the combined model (e.g. cluster-level GHF and individual level SES) cause some variables to drop out of the model. Nevertheless, there is also an added value to presenting different levels of indicators in a single model, as they do not necessarily overlap completely. In our final model, both individual- and cluster-level variables were significantly associated with HIV risk, indicating that both environmental factors at the cluster level and individual heterogeneity within the cluster are associated with increased HIV risk.

5. *Population attributability - it may be worth considering a Poisson model with robust variance to estimate relative risks and the leverage prevalence of exposure to various risk factors to estimate population attributable fractions for each in addition to the effect sizes currently presented. This may reveal some additional subtle differences across the countries and within. Additionally, a decomposition type approach (e.g. Shapley) using a generalised linear framework could also be used to estimate the relative importance of the various covariates.*

Thank you for this suggestion. We primarily decided to use logistic regression, since this is the most appropriate model for binary data. The reviewer is correct that our data can be approximated as count data, using a modified Poisson model. We ran the final fitted model as modified Poisson, and calculated relative risks (RRs). When the number of HIV tested people per location is large enough and overall prevalence levels are low, the logistic regression will approximate a Poisson, and odds ratios (ORs) and RRs will be relatively similar [20]. This is reflected in the outcomes (see S9 Table and S10 Table).

We decided to not provide population attributable fractions (PAFs), since they would incorrectly imply causality of the reported associations. Furthermore, some covariates, especially the sexual behavioural covariates, can act as mediators on the path from other covariates (i.e. education, socioeconomic status) to HIV. As a result, one would not be able to attribute risks to specific factors in the model.

We gave an explanation for the interpretation of the ORs from our models in the methods section, 5^th^ paragraph: “The final model is also fitted as modified Poisson (with robust variance) for easier interpretation of the estimates, here as relative risks (RRs) instead of odds ratios (ORs)”, and in the results section, 4^th^ paragraph: “The combined ‘full’ model was also fitted as modified Poisson regression model, where the aRRs were comparable to the aORs from the logistic regression model. A complete overview of the bivariate models, nested model (only adjusted for age and sex), best fitting models and final model as modified Poisson is provided in the supplementary information (S4 to S10 Tables and S7 Figure).”

6. *Results narrative - I think the explanation of the directionality of association for the environmental covariates e.g. EVI and GHF needs to be improved (e.g. lines 216-217).*

Agreed. We have revised our manuscript to improve the general explanation of the variables (in the methods) and improved our explanation of the directionality of the association (in the results).

Methods, 3^rd^ paragraph:

“EVI reflects the vegetation in an area, where low values represent areas with no or low green vegetation (e.g. big cities) and high values represent areas where vegetation is more abundant (e.g. forests, grassland), and can be used as proxy for degree of urbanization [9]. GHF represents the relative human influence and economic activity, incorporating nine layers covering infrastructure, land use, population density, and access (coastlines, roads, railroads, navigable rivers). Areas of large human influence and high levels of economic activity are characterised by high GHF values.”

Results, 4^th^ paragraph:

“Also, population density, proximity to nearest major city, EVI and GHF at location of a DHS cluster showed a significant association with HIV. […] HIV prevalence levels did not differ considerably between sample locations with different levels of greenness (indicated by the EVI). Living in an area with a relatively high GHF, as proxy for economic activity, was associated with a high HIV risk (aOR 1.68 [1.41; 1.94], p-value <0.001): HIV prevalence levels for the highest two levels of GHF were almost 7%, compared to around 4% at the lower levels.”

7. *If I understand Figure 3 correctly the least heterogeneity is explained in the areas with the highest prevalence among young adults (Figure 2)? This is important and has implication for the conclusions/utility of the results.*

This is partly correct. The interpolated random effect estimates demonstrate the unexplained heterogeneity. In figure 3A, we show that these are, as expected, especially high in high prevalence areas, as here the deviation from the mean observed prevalence is highest. However, we cannot conclude whether our covariates, introduced in the other models (Figure 3B to Figure 3E) are “better” at explaining heterogeneity in the low prevalence areas. In fact, we find that these variables explain heterogeneity in the high prevalence areas equally well, if not better, as random effect estimates in these areas decline more profoundly.

We explained this interpretation in more detail in the caption of Figure 3: “For the nested ‘empty’ model (A), random effect estimates reflect HIV prevalence levels among young adults (see Figure 2B). For the other models (B to E), random effect estimates are lower at (some of the) areas with high HIV prevalence levels, indicating that the additional variables in each model to some extent explain HIV heterogeneity at these locations.”

*Minor*

1. *Risk factor analysis: S2 Table - the coefficient for gender looks incorrect i.e. 0.00? Especially given the highly significant coefficient in S3 Table.*

Our notation was indeed confusing. The aOR the reviewer refers to is coded as 0.00 for women in this bivariate model describing the association between HIV and paid sexual intercourse, as ‘having paid for sex’ only applies for men in this dataset. We have now used N/A instead.

***** Reviewer #3:**

*This well-organized study uses geospatial methods to identify areas of high HIV prevalence among young adults within 7 countries in Eastern and sub-Saharan Africa. Additionally, the authors present a regression model that relates epidemiological predictors to HIV prevalence. While the approach is sound, the significance of is more of an incremental contribution rather than a major advance from published geospatial modelling studies of HIV prevalence, such as the works by Cuadros et al and Dwyer-Lindgren et al that are cited in the introduction of the paper. The focus on HIV prevalence among young adults is interesting and could be developed further.*

We thank the reviewer for her/his positive assessment of our manuscript.

*Major critiques:*

1. *The paper focuses on HIV prevalence among young adults, with the claim that young adults represent more recent infection and that high prevalence locations can be prioritized for prevention interventions. This is an interesting focus and could be developed further in the paper. Are the locations of high HIV prevalence in young adults the same as the locations among the general adult population? How do areas of high prevalence among young adults compare with variation in the underlying population age structure? More can be said in the discussion about efforts to prevent HIV infection specifically in this age group.*

We are happy that the reviewer acknowledges the importance of specifically looking at young adults for this analysis, and we appreciate her/his suggestions. This comment has some overlap with the 2^nd^ ‘General: …’ comment from reviewer #2. We added the following analyses to the supplementary material to check for (in)consistencies in interpolated HIV prevalence levels in various subgroups: “We compared the sex-specific surfaces of HIV prevalence by means of mapping the square root of the squared difference in HIV prevalence, to illustrate the absolute differences in HIV prevalence (per 5 km^2^ grid cell) (S6A Figure), and by plotting the predicted HIV prevalence (per 5 km grid cell) of women against the predicted HIV prevalence of men (S6C Figure). […] We repeated the same procedure to compare HIV prevalence levels between adults and young adults.”

We revised the text in the manuscript accordingly (results section, 2^nd^ paragraph):

“Overall, HIV prevalence is higher among adults, with high prevalence areas being in the same locations, but larger and more spread out compared to young adults (illustrated by the large red and purple areas).”

As per reviewers’ suggestion, we also added a section on prevention and treatment interventions to be specifically targeted at young adults (discussion, 4^th^ paragraph):

“Prevention programs aimed at improving exposure and uptake of effective prevention interventions for young adults [21,22] – such as pre-exposure prophylaxis (PrEP) [23], condoms [24], or voluntary male medical circumcision programmes [12,25] - should be prioritized in these areas. Also, intervention programs for young adults should aim at early diagnosis and treatment initiation of those who get infected with HIV, by creating accessible, affordable and youth-friendly HIV testing and counselling services [21,26]. Furthermore, governments typically have prior knowledge about future economic developments. Improving the resilience of affected populations against the associated health risks, might be considered an integral part of such development.”.

2. *Line 171-172 and Figure 1. How were the national-level prevalence estimates generated? Are these the weighted means of the cluster values or do they use the estimated HIV prevalence rasters? How do they compare with the published survey reports? It would enhance the results to provide a confidence interval around the estimate.*

We appreciate the reviewers’ comment, which is partly similar to minor comment #3 from reviewer #2. We originally did not incorporate the sample weights in our analyses, because we combined data of multiple countries and explored local level variances. We agree with the reviewer that the national-level HIV prevalence estimates should be representative and match the estimates provided in the official DHS and AIS reports. Therefore, we now incorporated the sample weights to calculate the national-level HIV prevalence estimates, as provided in Figure 1. We adapted the text regarding usage of sample weights in the methods section of the manuscript accordingly (see minor comment #3 of reviewer #2), see the first paragraph of the methods section: “Sample weights are incorporated in the DHS to translate unbalanced sampling into national representative data. In our study, we used these sample weights to estimate national HIV prevalence among adults and young adults for all seven countries. For the other analyses we combined data of multiple countries and explored local level variances, and thus not used sampling weights in our analyses.”

3. *The nine-year timespan over which the survey data were collected means that the resulting maps are a composite of various survey years. The years represented in the study coincided with a major scale-up of ART and HIV prevention interventions, so the difference in timing could be important. The difference in survey years should be included in the limitations.*

Good point, and we fully agree that this issue should be mentioned in the manuscript. Still, we do think that the potential bias introduced by the differences in survey year is limited. Although we know that the major scale-up of ART and HIV prevention interventions played major roles in curbing the epidemic, and potentially even disproportionally at high transmission areas, we expect that the disproportionate impact would not be so extreme that it completely altered the locations of high transmission areas.

We expect that ART would first affect the longevity of the patients accessing it, affecting incidence levels and then prevalence among young adults with a delay. This is reflected in the data; in Zambia, for instance, where ART scale-up took place between 2001 and 2005 approximately, HIV prevalence among young adults (below 25 years of age) dropped among women, but increased slightly among men. This suggests that in this period ART seems to have a marginal impact on HIV transmission among young adults, which in fact happens to be in line with the somewhat sobering finding from the field trials on Treatment as Prevention (TasP).

We added the following to the limitation section of the discussion (5^th^ paragraph):

“… we used survey data from a relatively wide range of years; 2008-2009 to 2015-2016. This time period in the study coincides with major initiatives to curb the pandemic, in particular the scale-up of antiretroviral treatment, as well as voluntary male circumcision campaigns and other HIV prevention interventions. Although these initiatives were potentially disproportionally targeted at high transmission areas, we expect that the disproportionate impact would not be so extreme that it completely alter the locations of high transmission areas. Furthermore, although the scale-up of these interventions may have reduced HIV incidence, the associations between HIV and the hypothesised main drivers of HIV transmission – the (sexual) behavioural, socioeconomic and environmental factors explored in this study – likely did not change substantially by these interventions.”

4. *The claim in the discussion lines 278-279 that all high prevalence areas were areas with known high levels of economic activity needs to be tested more rigorously. How is the level of economic activity for an area defined in this analysis? Was the level of economic activity assessed over the entire geographic area? Were areas also found that had high economic activity and low HIV prevalence?*

Locations of economic activity are defined as “[areas that are] characterised by high production and flow of goods and services”, now also clearly stated in the manuscript (methods, 5^th^ paragraph). The locations of these areas of economic activity - in SSA mainly characterized by mining, fishing, and truck-driving at border crossings - and their association with higher HIV risk, are well-known from previous studies [27–32]. We identified these areas (indicated by the circles in Figure 3 of the study) and compared them to the areas with high levels of HIV transmission, as identified in our study. These locations to a large extent overlap. So, we did not use one measure for economic activity, but rather used information from existing literature to identify these areas.

*Minor critiques*

1. *Please make it clear which years are represented for the covariates that were not directly extracted from the DHS or AIS surveys (e.g. WorldPop).*

As per reviewers’ suggestion, we added the years of the population density, enhanced vegetation index and global human footprint estimates to the ‘methods’ section, ‘data’ subsection: “[…] data from 2010, the most recent year, were utilised.” and “EVI data were available for 2010 and GHF for 2005.”

2. *Include a justification for why a larger age group was defined as young adults for males than for females.*

We chose to use different cut-offs for men and women, based on the knowledge that men often have their sexual debut at an older age than women, and often have sexual relationships with younger women. We have added the following sentence to the methods section, 2^nd^ paragraph: “The discrepancy in age cut-off reflects the common age-difference in sexual debut and relationships between women and men [33].”

3. *Line 124 statistical analysis. Please provide the interpolation equations in the supplementary material.*

We have added the ordinary kriging and semivariance equations to the supplementary material (S1 Equations) and referred to the supplementary material in the first paragraph of the ‘statistical analyses’ subsection of the methods section: “The equations and model estimates are provided in the supplementary information (S1 Equations)”.

4. *How does the amount of variation explained by the models in this study compare to R^2^ in other published studies?*

This is an interesting suggestion. We searched the literature, but found no studies that explicitly mention the R^2^ from comparable models. The explained heterogeneity (R^2^) highly depends on the type of model, number of covariates, type of covariates and the overall scale of the study. We explored many behavioral covariates and used one model to predict HIV status for people from 7 different countries, with many other varying factors that we did not incorporate in our model. Therefore, the R^2^ are according to what we would expect from this type of analysis.

5. *Line 300. I do not agree that the findings of this study support the need to increase condom use specifically. Rather, persistently high HIV incidence supports the need to increase access to effective HIV prevention interventions more broadly.*

We agree with the reviewer and have changed this statement to “We found that the reported condom use at last sex act was associated with an increased risk of being infected with HIV. This is a well-known counterintuitive finding, and reflects […] This finding highlights the need to stimulate condom use, but more importantly increase access to effective HIV prevention interventions more broadly.” (discussion, 3^rd^ paragraph).

6. *Line 317, "We are the first…". Please modify this statement in light of the other published HIV prevalence maps cited in the paper. While this paper's focus on young adults takes a new lens to these data, they are fully utilized in the other studies as well.*

We agree with the reviewer and changed the sentence in line with the reviewers’ feedback, to “To our knowledge, we are the first to utilise geolocated HIV prevalence data from young adults specifically, to explore geospatial heterogeneity in the HIV epidemics of Eastern and Southern Africa, and identify potential transmission areas.” (discussion section, first sentence of the 5^th^ paragraph).

**References**

1.	Von Elm E, Altman DG, Egger M, Pocock SJ, Gøtzsche PC, Vandenbroucke JP, et al. The Strengthening the Reporting of Observational Studies in Epidemiology (STROBE) Statement: guidelines for reporting observational studies. Int J Surg. 2014;12: 1495–1499.

2.	Palk L, Blower S. Geographic variation in sexual behavior can explain geospatial heterogeneity in the severity of the HIV epidemic in Malawi. BMC Med. 2018;16: 22. doi:10.1186/s12916-018-1006-x

3.	Bachanas P, Medley A, Pals S, Antelman G, Benech I, Deluca N, et al. Disclosure, Knowledge of Partner Status, and Condom Use Among HIV-Positive Patients Attending Clinical Care in Tanzania, Kenya, and Namibia. AIDS Patient Care STDS. 2013;27: 425–435. doi:10.1089/apc.2012.0388

4.	Neve J De, Fink G, Subramanian S V, Moyo S, Bor J. Length of secondary schooling and risk of HIV infection in Botswana: evidence from a natural experiment. Lancet. 2015;3: 470–477. doi:10.1016/S2214-109X(15)00087-X

5.	Baird SJ, Garfein RS, McIntosh CT, Özler B. Effect of a cash transfer programme for schooling on prevalence of HIV and herpes simplex type 2 in Malawi: A cluster randomised trial. Lancet. 2012;379: 1320–1329. doi:10.1016/S0140-6736(11)61709-1

6.	Steyerberg EW, Harrell FE. Prediction models need appropriate internal, internal--external, and external validation. J Clin Epidemiol. 2016;69: 245–247.

7.	Steyerberg EW. Validation in prediction research: the waste by data splitting. J Clin Epidemiol. 2018;103: 131–133.

8.	Gasser T, Sroka L, Jennen-Steinmetz C. Residual variance and residual pattern in nonlinear regression. Biometrika. 1986;73: 625–633.

9.	Cuadros DF, Li J, Branscum AJ, Akullian A, Jia P, Mziray EN, et al. Mapping the spatial variability of HIV infection in Sub-Saharan Africa: Effective information for localized HIV prevention and control. Nature. 2017;7: 1–11. doi:10.1038/s41598-017-09464-y

10.	Tanser F, LeSueur D, Solarsh G, Wilkinson D. HIV heterogeneity and proximity of homestead to roads in rural South Africa: An exploration using a geographical information system. Trop Med Int Heal. 2000;5: 40–46. doi:10.1046/j.1365-3156.2000.00513.x

11.	Barankanira E, Molinari N, Niyongabo T, Laurent C. Spatial analysis of HIV infection and associated individual characteristics in Burundi: Indications for effective prevention. BMC Public Health. 2016;16: 1–11. doi:10.1186/s12889-016-2760-3

12.	Auvert B, Taljaard D, Lagarde E, Sobngwi-Tambekou J, Sitta R, Puren A. Randomized, controlled intervention trial of male circumcision for reduction of HIV infection risk: The ANRS 1265 trial. PLoS Med. 2005;2: 1112–1122. doi:10.1371/journal.pmed.0020298

13.	Merlo J, Chaix B, Ohlsson H, Beckman A, Johnell K, Hjerpe P, et al. A brief conceptual tutorial of multilevel analysis in social epidemiology: Using measures of clustering in multilevel logistic regression to investigate contextual phenomena. J Epidemiol Community Health. 2006;60: 290–297. doi:10.1136/jech.2004.029454

14.	Wilson D, Halperin DT. “Know your epidemic, know your response”: a useful approach, if we get it right. Lancet. 2008;372: 423–426. doi:https://doi.org/10.1016/S0140-6736(08)60883-1

15.	United Nations. Transforming our World: The 2030 Agenda for Sustainable Development. [Internet]. United Nations Division for Social Policy and Development, Indigenous Peoples; 2017. Available: https://sustainabledevelopment.un.org/post2015/

16.	UNAIDS. Fast-track: ending the AIDS epidemic by 2030. 2014.

17.	Reniers G, Wamukoya M, Urassa M, Nyaguara A, Nakiyingi-Miiro J, Lutalo T, et al. Data resource profile: network for analysing longitudinal population-based HIV/AIDS data on Africa (ALPHA Network). Int J Epidemiol. 2016;45: 83–93.

18.	Dwyer-Lindgren L, Cork MA, Sligar A, Steuben KM, Wilson KF, Provost NR, et al. Mapping HIV prevalence in sub-Saharan Africa between 2000 and 2017. Nature. 2019; doi:10.1038/s41586-019-1200-9

19.	Dwyer-lindgren L, Cork MA, Sligar A, Steuben KM, Wilson KF, Provost NR, et al. Mapping HIV prevalence in sub-Saharan Africa between 2000 and 2017. Nature. 2019; doi:10.1038/s41586-019-1200-9

20.	McNutt L-A, Wu C, Xue X, Hafner JP. Estimating the relative risk in cohort studies and clinical trials of common outcomes. Am J Epidemiol. 2003;157: 940–943.

21.	Mavedzenge SMN, Doyle AM, Ross DA. HIV prevention in young people in sub-Saharan Africa: a systematic review. J Adolesc Heal. 2011;49: 568–586.

22.	Pettifor A, Bekker L-G, Hosek S, DiClemente R, Rosenberg M, Bull S, et al. Preventing HIV among young people: research priorities for the future. J Acquir Immune Defic Syndr. 2013;63: S155.

23.	Baeten JM, Donnell D, Ndase P, Mugo NR, Campbell JD, Wangisi J, et al. Antiretroviral Prophylaxis for HIV Prevention in Heterosexual Men and Women. N Engl J Med. 2012;367: 399–410. doi:10.1056/NEJMoa1108524

24.	Creese A, Floyd K, Alban A, Guinness L. Cost-effectiveness of HIV / AIDS interventions in Africa : a systematic review of the evidence. Lancet. 2002;359: 1635–1642.

25.	Samuelson J, Dickson K. Progress in scale-up of male circumcision for HIV prevention in Eastern and Southern Africa: Focus on service delivery. World Health Organization and UNAIDS. 2011.

26.	Wong VJ, Murray KR, Phelps BR, Vermund SH, McCarraher DR. Adolescents, young people, and the 90-90-90 goals: a call to improve HIV testing and linkage to treatment. AIDS. 2017;31: S191.

27.	Chang LW, Health R, Program S, Program BI, Hopkins J, Grabowski MK, et al. Heterogeneity of the HIV epidemic: an observational epidemiologic study of agrarian, trading, and fishing communities in Rakai, Uganda. Lancet HIV. 2016;3: 1–20. doi:10.1016/S2352-3018(16)30034-0.Heterogeneity

28.	Tumwesigye NM, Atuyambe L, Wanyenze RK, Kibira SP, Li Q, Wabwire-Mangen F, et al. Alcohol consumption and risky sexual behaviour in the fishing communities: Evidence from two fish landing sites on Lake Victoria in Uganda. BMC Public Health. 2012;12: 1–11. doi:10.1186/1471-2458-12-1069

29.	Cassels S, Camlin CS. Geographical mobility and heterogeneity of the HIV epidemic. Lancet HIV. 2016;3: e339–e341. doi:10.1016/S2352-3018(16)30048-0

30.	Faria NR, Rambaut A, Suchard MA, Baele G, Bedford T, Ward MJ, et al. The early spread and epidemic ignition of HIV-1 in human populations. Science (80- ). 2014;346: 56–61.

31.	Corno L, Walque D De. Mines , Migration and HIV / AIDS in Southern Africa. J Afr Econ. 2012;21: 465–498. doi:10.1093/jae/ejs005

32.	Clift S, Anemona A, Watson-Jones D, Kanga Z, Ndeki L, Changalucha J, et al. Variations of HIV and STI prevalences within communities neighbouring new goldmines in Tanzania: importance for intervention design. Sex Transm Infect. 2003;79: 307 LP – 312.

33.	Luke N, Kurz K. Cross-generational and transactional sexual relations in sub-Saharan Africa. Washington, DC Int Cent Res Women. 2002;
