# Supplementary material for: Mapping and characterising areas with high levels of HIV transmission in sub-Saharan Africa: A geospatial analysis of national survey data
Source: PLoS Med. 2020 Mar 6;17(3):e1003042. doi: 10.1371/journal.pmed.1003042 (PMC7059914; doi:10.1371/journal.pmed.1003042)
Supplement: Attachment — Submitted filename: Response to the requests from the editors FINAL.docx [file pmed.1003042.s029.docx]

*1.	In the abstract, please present numbers in the format of "113,000 adults" at line 32, for example.*

This has been changed.

*2.	Around line 33, again in the abstract, we suggest adding a sentence to quote the range of mean levels of HIV prevalence by country, for adults and young adults, to contrast the local numbers quoted subsequently.*

We added the following sentence to methods section of the abstract: “National-level HIV prevalence for young adults ranged from 2.2% in Tanzania to 7.7% in Mozambique. However, at the subnational level we found areas with prevalence among young adults as high as 11% or 15% alternating with areas with prevalence between 0% and 2%, suggesting the existence of areas with high levels of transmission”

*3.	Also, at lines 32 and 247, please make that "... of which about 53,000 were young adults ..." or similar.*

This has been adapted.

*4.	At line 35, we suggest adapting the current wording to " ... among young adults as high as 11% or 15%"*

This has been changed, the new sentence is provided under comment 2.

*5.	At line 59, the wording "Heterogeneity could be explained for 15.6% ..." suggests "15.6 of participants", and we ask you to reword this text if you are referring to "15.6% of heterogeneity" (and similarly at line 367).*

We agree with the editors that these sentences in de abstract and discussion needed to rephased, and adapted the text according to the editors’ suggestion.

Abstract:

“Overall, 15.6% of heterogeneity could be explained by an interplay of known behavioural, socioeconomic and environmental factors.”

Discussion section:

“Overall, 15.6% (marginal R^2^) of the heterogeneity in HIV prevalence could be explained by an interplay of behavioural, socioeconomic and environmental factors, including number of sex partners, STIs, global human footprint (a proxy for economic activity), and urbanization.”

*6.	In the abstract, you may wish to quote additional findings from column 3 of table 2 in a new sentence, say, to help readers appreciate the context of the quoted value of "15.6%".*

Thank you for this suggestion. The findings from this column are already explained in words in the sentence that follows: “Maps of the interpolated random effect estimates show that environmental variables, representing indicators of economic activity, were most powerful in explaining high prevalence areas.”. We decided to leave this part as it is and not add more specific numbers, because we believe the message is more easily understandable for the broader public of the journal this way. We do mention and describe these details in the main text.

*7.	At line 42, please make that "main study limitations".*

Done.

*8.	Should "eSwatini" be substituted at line 90?*

Correct, we changed this to “eSwatini (former Swaziland)”.

*9.	At line 239, please make that "followed RECORD guidelines".*

This has been changed.

*10.	At line 316, should that be "7.2% (marginal R^2^) or 26.3% (conditional R^2^) of the HIV heterogeneity ..."?*

We thank the editors for this suggestion. The marginal R^2^ falls within the conditional R^2^, and therefore the current phrasing is correct. To enhance readability, we now added descriptions of the two types of presented R^2^; “The results in Table 1 show that, looking at the nested ‘empty’ model, 7.2% (marginal R^2^: e.g. percentage of heterogeneity explained by fixed effects in the model) of the 26.3% (conditional R^2^: e.g. percentage of heterogeneity explained by both fixed and random effects in the model) of the HIV heterogeneity could be explained by age and sex alone.”.

*11.	At line 320, please add a comma ("... sexual, behavioural ...").*

We agree that this sentence is not clear, the word "sexual" is removed now.

*12.	At line 363, please make that "substantial levels ... exist among ...".*

“exists” has been changed to “exist”

*13.	At line 368, please add a few words to explain what "global human footprint" is.*

Thank you for noting this, we added the following description: “global human footprint (a proxy for economic activity)”

*14.	At line 453, please amend the text to "... DHS surveys produce cross-sectional data intended to provide a measure of HIV prevalence and behaviour ..." or similar.*

We like the suggestion of the editors and adapted this sentence in the discussion section accordingly: “Our results have limitations. First, DHS surveys produce cross-sectional data intended to provide a measure of HIV prevalence and behaviour among the general population; and thus, high-risk subpopulations such as female sex workers, men who have sex with men, and mobile populations such as truck drivers and seasonal workers, are thought to be underrepresented in these surveys.”

*15.	Please add fuller access details to reference 11, as available.*

All available information has now been added:

“Luke N, Kurz K. Cross-generational and transactional sexual relations in sub-Saharan Africa. Washington, DC, International Center for Research on Women. 2002;1, 1–42.”

*16.	Please remove "[Internet]" from reference 16 and any other relevant references, and add a cited date.*

Thank you for noting, this has been changed for this reference and elsewhere.

*17.	Please convert the RECORD checklist to a separate supplementary file, named "S1_Checklist".*

Done.
